# Live-attenuated *Toxoplasma gondii* RHΔ*ddx6* mutant establishes comprehensive immunity against acute, chronic, and congenital toxoplasmosis

**DOI:** 10.1080/21505594.2026.2713823

**Published:** 2026-08-07

**Authors:** Zhenke Yang, Jinghui Wang, Junru Wu, Youke Fan, Tingting Ying, Haina Zhang, Jiakang Bai, Yuanfeng Wang, Ping Liu, Guofeng Li, Mengxue Nie, Xiaowei Tian, Xuefang Mei, Zhenchao Zhang, Qiangqiang Wang, Shuai Wang

**Affiliations:** a Xinxiang Key Laboratory of Pathogenic Biology, Department of Pathogenic Biology, School of Basic Medical Sciences, Henan Medical University, Xinxiang, China; b Department of Rehabilitation, The First Affiliated Hospital of Henan Medical University, Xinxiang, China; c College of Animal Science and Veterinary Medicine, Henan Institute of Science and Technology, Xinxiang, China

**Keywords:** *T. gondii*, DDX6, live-attenuated vaccine, immunogenicity, congenital protection

## Abstract

*Toxoplasma gondii* is an obligate intracellular parasite causing severe disease in immunocompromised individuals and congenitally infected infants. Despite decades of research, no licensed human vaccine exists. This study evaluates a novel live-attenuated vaccine candidate based on depletion of DDX6, a conserved DEAD-box RNA helicase involved in post-transcriptional gene regulation. A Δ*ddx6* strain was generated in the type I virulent RH strain using CRISPR/Cas9, and its phenotype was comprehensively assessed. Although the mutant displayed only subtle defects in certain *in vitro* assays, it exhibited almost complete avirulence in mice even at high inoculums (10^6^ tachyzoites), representing a dramatic attenuation phenotype not fully predicted by *in vitro* analyses. A single immunization with 1 × 10^6^ Δ*ddx6* tachyzoites induced robust Th1-biased immunity characterized by high *T. gondii*-specific IgG titers, CD4^+^/CD8^+^ T-cell activation, and IFN-γ production. Vaccinated mice achieved 100% survival against lethal RH challenge and showed a 99.5% reduction in brain cyst burden following PRU strain infection. Furthermore, the vaccine completely prevented vertical transmission in a pregnancy model. Transcriptomic analysis revealed widespread dysregulation in the Δ*ddx6* strain, particularly downregulation of key virulence factors (ROPs, SAGs) and genes involved in intracellular transport. These findings demonstrate that targeting DDX6 generates a highly attenuated yet strongly immunogenic strain with exceptional cross-stage protective efficacy against acute, chronic, and congenital toxoplasmosis, representing a promising vaccine candidate worthy of further development.

## Background

*Toxoplasma gondii* is an obligate intracellular apicomplexan parasite with a remarkably broad host range, capable of infecting virtually all warm-blooded animals [1]. This cosmopolitan parasite poses a significant global public health burden, with seroprevalence rates varying dramatically across different regions, estimated to affect approximately one-third of the human population worldwide [2]. Human infection occurs primarily through the ingestion of tissue cysts present in undercooked meat or through the accidental consumption of oocysts shed by infected felids, which contaminate soil and water [3].

While infection in immunocompetent individuals is typically asymptomatic or presents as a self-limiting influenza-like illness, it can lead to severe, life-threatening pathology in immunocompromised hosts and pregnant women. In individuals with compromised cellular immunity, such as those with AIDS or recipients of immunosuppressive therapy, reactivation of latent infection can cause devastating sequelae, most notably toxoplasmic encephalitis [4]. One of the most severe consequences of *T. gondii* infection is linked to primary infection during pregnancy, due to the risk of vertical transmission and congenital toxoplasmosis. This condition can result in spontaneous abortion, stillbirth, or a wide spectrum of severe neurological and ocular manifestations in the newborn, including hydrocephalus, intracranial calcifications, and chorioretinitis. Furthermore, the timing of maternal infection is a critical determinant of outcome; while transmission rates are lower in the first trimester, the resulting fetal disease is often more severe. Consequently, *T. gondii* represents a significant perinatal and teratogenic concern, underscoring the urgent need for effective preventive strategies [5,6].

Decades of research have explored a diverse array of vaccine platforms against toxoplasmosis. Initial efforts focused on killed or inactivated whole-parasite vaccines but yet demonstrated inadequate protection, primarily inducing humoral immunity with poor cellular responses [7]. Subunit vaccines, utilizing recombinant proteins such as surface antigens (SAGs) and dense granule proteins (GRAs), have shown promise by eliciting partial protection in mouse models [8]. DNA vaccines and, more recently, nanoparticle-based and mRNA vaccine formulations have been investigated to enhance the immunogenicity of key antigens [9,10]. Despite these extensive efforts, no vaccine has been approved for human use to date. The challenges include the genetic diversity of *T. gondii* strains, the complexity of its life cycle involving multiple stages (tachyzoites, bradyzoites, oocysts), and the critical need to stimulate a robust and balanced T-helper 1 (Th1)-type cellular immune response, characterized by interferon-gamma (IFN-γ) production, for effective control of intracellular replication [11].

Among all strategies, live-attenuated vaccines have consistently demonstrated superior efficacy, as they most closely mimic natural infection by effectively presenting the full repertoire of parasite antigens and promoting strong, durable cellular immunity [12]. The S48 strain, developed through prolonged passaging and commercialized as Toxovax® for veterinary use in sheep, is a testament to the potential of this approach [13]. However, concerns regarding potential reversion to virulence and the residual ability to form tissue cysts have limited its broader application [14]. The advent of precise genetic engineering tools, particularly the CRISPR/Cas9 system, has revolutionized this field [15]. It has been successfully applied to construct attenuated strains of *T. gondii* by disrupting specific virulence-associated genes, such as those encoding dense granule proteins like GRA72 [16] or rhoptry proteins like ROP18 [17], as well as genes involved in essential metabolic pathways like 6-phosphogluconate dehydrogenase (6PGDH1) [18] and succinyl-CoA synthetase (SCSA) [19]. Although monogenic knockout strains can be attenuated and elicit protective immunity, their safety profile may be compromised by the theoretical risk of virulence reversion through compensatory mutations. Therefore, identifying critical virulence factors, the deletion of which leads to a stably attenuated phenotype, is crucial for developing next-generation vaccines with an enhanced safety profile.

In this pursuit, we focused on targeting global regulators of parasite biology. The DEAD-box family of RNA helicases, characterized by their conserved Asp-Glu-Ala-Asp (DEAD) motif, play fundamental roles in RNA metabolism and have been implicated in microbial stress adaptation and virulence regulation [20–23] (Figure S1). Among them, DDX6 is a key regulator of post-transcriptional gene expression, operating within processing bodies (P-bodies) to control mRNA stability and translation [24,25]. Despite its well-established role in eukaryotic cellular processes, the function of DDX6 in *T. gondii* biology and pathogenesis remains uncharacterized. Given the central role of DDX6 in post-transcriptional gene regulation across eukaryotes, we sought to investigate its function in the biology of *T. gondii*, an obligate intracellular parasite where precise control of gene expression is likely critical for survival and pathogenesis. To this end, a DDX6-knockout strain (Δ*ddx6*) was successfully constructed in the virulent RH background using CRISPR/Cas9 gene editing. We then conducted a comprehensive functional characterization of the mutant strain, which included evaluating its *in vitro* growth and replication phenotypes, assessing its attenuated virulence profile in mouse models, and systematically testing its efficacy as a live-attenuated vaccine candidate against challenges mimicking acute, chronic, and congenital toxoplasmosis. Consistently, the Δ*ddx6* strain demonstrated severe virulence attenuation and conferred sterilizing immunity across all challenge models, establishing it as a highly promising and safe vaccine candidate.

## Materials and methods

### Animals and cells

All 6-week-old (22-25 g weight) SPF-grade ICR mice were purchased from SPF (Beijing) Biotechnology Co., Ltd. and were raised in the specific pathogen-free environment of Henan Medical University. They were fed with sterile feed and purified water under suitable temperature and humidity conditions, and group assignment was randomized by Excel’s RANDBETWEEN function. Investigators performing animal randomization, preparation of injectants, immunization, challenge infection, and routine monitoring were aware of group allocation due to the nature of the experimental procedures. However, brain cyst counting and histopathological evaluations were performed in a blinded manner to minimize potential bias. Specifically, the investigators responsible for cyst quantification and histopathological evaluation were not informed of the group allocation during sample analysis, and group information was revealed only after completion of measurements and data recording.

Sample sizes for animal experiments were determined based on expected effect sizes from preliminary observations. For survival experiments, group sizes of 10 mice were selected to detect a large protective effect, assuming an expected survival difference of approximately 70–100% between vaccinated and control groups. For parasite burden, cyst counting, and immunological assays, group sizes ranging from 3 to 10 animals were selected according to the expected biological variability and anticipated magnitude of differences between groups. For the congenital infection model, 30 female mice per group were initially allocated to account for mating success, pregnancy establishment, and potential pregnancy loss after challenge; fetuses and placentas from successfully pregnant dams were subsequently analyzed as the experimental outcomes. All sample sizes were selected while minimizing animal use in accordance with ethical principles. All the experiments in this study were approved by the Ethics Review Committee of Xinxiang Medical University (reference number: XYLL-20230093). At the end of the experiments, mice were euthanized by cervical dislocation, a method that swiftly dislocates the cervical spine, resulting in immediate death and minimizing potential pain and distress, in accordance with animal welfare guidelines. The *T. gondii* RH Δ*ku80* (hereinafter referred to as RH) and PRU strains used in this study have been preserved by Xinxiang Key Laboratory of Pathogenic Biology. RH strains proliferate in human foreskin fibroblast (HFF) cells (SCRC-1041, ATCC, USA). For the PRU strain, its subculture was achieved by oral inoculation of cysts into mice.

### Generation of the Δddx6 by CRISPR/Cas9

The experimental procedures for genetic manipulation were performed as described by Shen et al. [15]. The *ddx6* gene (Gene ID: TGGT1_313010) was disrupted in *T. gondii* RH strain tachyzoites via CRISPR/Cas9-mediated homologous replacement of its entire coding sequence with a pyrimethamine-resistant DHFR-TS selection cassette. Two specific sgRNAs were designed using the E-CRISPR online tool (http://www.e-crisp.org) with strict parameters (NGG PAM, 5’ G base) targeting early exons, based on the gene sequence obtained from ToxoDB (https://toxodb.org/toxo/app). The pSAG1:Cas9-U6:sgddx6 plasmid, harboring both sgRNA expression cassettes and the homologous recombination template, was constructed and introduced into parasites by electroporation. Positive clones were selected under 3 μM pyrimethamine pressure and successful gene knockout was confirmed by diagnostic PCR and subsequent Sanger sequencing. All primers and sequences used in this study are listed in Table 1.

**Table 1.** Primer sequence table used in the experiment.

Name	Sequence (5’→3’)
*ddx6*-sgRNA1	GGGTTGTCTTCTGTGTGTCC
*ddx6*-sgRNA2	GCTGTTCTTGTGGCCAAGTG
Δ*ddx6*-PCR1	F: GCACAATTAGCCATCAGCTTCACC R: CTTAATGTCCACGTAGTTCGCGC
Δ*ddx6*-PCR2	F: CGATTGGCGATACGTCTCTGTTGA R: ATGTATCGGAAGTCAGCTGAGCAC
Δ*ddx6*-PCR3	F: TACACGTTTGGGAGCAGAAACCCT R: AGAGTACATGCACCGGGTTGTACA
ROP4	F: CTCTCTCCGCCAAACTCCA R: CCGCTCATATCTGCCACCT
ROP8	F: AGCTAGGCGGAATTGAATGG R: GATACAGGGGCAAAACGGCT
ROP15	F: AAAGTCTGGAAGCCCTGGTAGA R: CGGCTCCCTGTCGCTTGTGG
ROP19A	F: TTGAGTCATTATTCAGCGGAGG R: AGGCTTCTTAGGCGTTGTAGATT
ROP24	F: CGAGAATCAGGAGTACGCTTTG R: CGTGATGCGGAGAAACAGG
ROP39	F: CGATTGTCAAGTGACGAACGA R: ATGGGACTGCGAACCGAA
SUB1	F: CAGCCCTATGCGGAGAAGA R: TTCTACTTTGCCTTGCAAAA
SUB3	F: TATGTGAAGTCAGGCGTAG R: CATTATGGGCTTGAGGAG
TgB1	F: TCCCCTCTGCTGGCGAAAAGT R: AGCGTTCGTGGTCAACTATCGATTG
mActin	F: GATGCAGAAGGAGATTACTG R: ACCGATCCACACAGAGTA
FOXP3	F: ACCATTGGTTTACTCGCATGT R: TCCACTCGCACAAAGCACTT
RORγt	F: TTGAAGGCAAATACGGTGGTGTG R: AGGGCAATCTCATCCTCAGAAAAAC

### Plaque assay

The plaque assay was conducted to evaluate the lytic capability of T. gondii tachyzoites (RH and Δ*ddx6* strains) by quantifying plaque formation on host cell monolayers. Prior to the assay, HFF monolayers were prepared in 6-well culture plates, with three replicate wells established for each experimental group. Healthy tachyzoites were inoculated onto the HFF cells at a density of 200 parasites per well, followed by stationary incubation at 37°C under 5% CO_2_ for 5–7 days. Plaque formation was monitored daily from day 5 post-inoculation. When plaques became clearly visible, the wells were gently washed three times with PBS, fixed with 4% paraformaldehyde for 15 minutes at room temperature, and washed again to remove residual fixative. The cells were then stained with 0.5% crystal violet solution for 60 minutes in the dark, after which excess stain was removed and the wells were rinsed with deionized water. After air-drying, high-resolution images of the plates were acquired using a flatbed scanner, and plaque number and area were quantified with ImageJ software (version 1.53, National Institutes of Health, USA). The experiment included three independent biological replicates to ensure reproducibility.

### Invasion assay

The invasion assay was performed to compare the host cell invasion efficiency of RH and Δ*ddx6* tachyzoites. Prior to infection, HFF monolayers were prepared in T25 culture flasks, with a blank HFF control included to account for background signal. RH and Δ*ddx6* tachyzoites were harvested after 48 hours of culture, washed with PBS to remove debris, and counted. Parasite numbers were adjusted to ensure consistent inoculum sizes across groups. Tachyzoites were labeled with CFDA-SE (GLPBIO, YGC14056) according to the manufacturer’s protocol: a 5 mM/mL stock solution was diluted 1:1000 in serum-free DMEM to a working concentration of 5 μM/mL, and parasites were incubated in the solution at 37°C with shaking for 30 minutes. Excess dye was removed by three washes with serum-free DMEM. Labeled parasites were resuspended and quantified by fluorescence intensity to ensure uniform labeling. A standardized number of tachyzoites (1 × 10^5^) was added to confluent HFF monolayers in 24-well plates and incubated at 37°C with 5% CO_2_ for 1 hour to permit invasion. Non-invaded parasites were removed by three gentle washes with PBS. Fresh complete medium was added, and the cultures were continued for 12 hours. Cells were then trypsinized, collected by centrifugation at 2000 g for 10 minutes, and resuspended in 100 μL PBS. The proportion of infected HFF cells (CFSE-positive) was analyzed by flow cytometry using the FITC channel. Each experiment included three technical replicates and was independently repeated three times.

### Proliferation assay

To assess intracellular replication capacity, HFF monolayers prepared in 6-well plates were infected with freshly egressed RH or Δ*ddx6* tachyzoites at a multiplicity of infection (MOI) of 1:1 (1 × 10^5^ parasites per well) and incubated at 37°C under 5% CO_2_ for 1.5 hours to synchronize invasion. Extracellular parasites were removed by three washes with pre-warmed PBS, and infected cells were further cultured for 24 hours. Subsequently, cells were fixed with 4% paraformaldehyde, stained with Wright-Giemsa solution, and examined microscopically. For each replicate, 200 parasitophorous vacuoles (PVs) were randomly selected under a 40× objective, and the number of tachyzoites per PV was quantified and categorized (1, 2, 4, 8, 16, or ≥32 parasites per vacuole). The proportion of PVs in each category was calculated to determine replication rates. The experiment was independently repeated three times, and data are presented as mean ± standard deviation (SD) [26].

### Gliding assay

The gliding motility assay was performed to compare the migratory capabilities of wild-type RH and Δ*ddx6* tachyzoites using a modified protocol based on established methods [27]. Sterile coverslips placed in a 24-well plate were coated with 300 μL of PBS containing 50 μg/mL BSA by incubation at 37°C for 30 minutes or at 4°C overnight. After removing the coating solution, the coverslips were washed three times with pre-warmed PBS and equilibrated with extracellular buffer (EC buffer: 5 mM KCl, 142 mM NaCl, 1.8 mM CaCl_2_, 1 mM MgCl_2_, 5.6 mM D-glucose, 25 mM HEPES, pH 7.4) at 37°C. Tachyzoites of both strains were harvested from freshly egressed cultures by mechanical disruption using a 22–25 G syringe needle, filtered through a 4 μm membrane, and centrifuged at 400–800 ×g for 10 minutes. The parasite pellets were resuspended in EC buffer and adjusted to a concentration of 1 × 10^7^/mL. A 500 μL aliquot (containing 5 × 10^6^ parasites) was inoculated onto the BSA-coated coverslips, followed by centrifugation at 200 ×g for 1 minute to synchronize contact with the substrate. The plates were incubated at 37°C with 5% CO_2_ for 15 minutes to allow gliding. After discarding the suspension, the coverslips were washed with PBS, fixed with 4% paraformaldehyde for 15 minutes, and blocked with 10% FBS-PBS. Samples were incubated with anti-SAG1 primary antibody (Thermo Scientific, MA518268) diluted in 1% FBS-PBS, followed by appropriate secondary antibodies and DAPI-containing mounting medium. Gliding trails and patterns were visualized and quantified using laser scanning confocal microscopy, and the length of gliding was quantified with ImageJ software.

### Egress assay

To evaluate the egress capacity of *T. gondii* tachyzoites, confluent HFF monolayers were prepared in 6-well plates, with three replicate wells per experimental group. Freshly harvested tachyzoites of the wild-type RH strain and the Δ*ddx6* mutant were inoculated into the wells at a dose of 1 × 10^5^ parasites per well. The plates were incubated at 37°C under 5% CO_2_ for 1–2 h to permit invasion, after which non-invaded parasites were removed by washing with phosphate-buffered saline (PBS). When plaques became visible in the host cell layer, 200 PVs per well were randomly examined using a 40× inverted microscope, and the proportion of spontaneously egressed parasites was recorded prior to stimulation. The cells were then treated with 3 μM calcium ionophore A23187 (Glpbio, #GC1120) for 5 min at 37°C to trigger induced egress. The stimulant was promptly removed, and the cells were fixed with 4% paraformaldehyde in PBS for 15 min at room temperature. The percentage of PVs showing egress after ionophore treatment was determined by counting 200 PVs per well. Data were obtained from three independent experiments.

### In vivo virulence assay of the T. gondii Δddx6 strain

The *in vivo* virulence of the wild-type RH strain and the Δ*ddx6* mutant was evaluated using a murine model. Fifteen mice per subgroup were intraperitoneally inoculated with 1 × 10^3^, 1 × 10^4^, or 1 × 10^6^ freshly harvested tachyzoites of either the RH strain (control groups) or the Δ*ddx6* strain (experimental groups). Following infection, mice were monitored every 12 hours for survival and clinical signs using a 10-point progressive scoring system (0: no abnormalities; 10: severe multisystem pathology). Assessed parameters included hunching, piloerection, ptosis, sunken eyes, worm-seeking behavior, latency of movement, ataxia, touch reflexes, deficient evacuation, and lying on belly. On day 7 post-infection, five randomly selected mice per subgroup were euthanized, and heart, liver, spleen, brain, and peripheral blood samples were aseptically collected. Tissues were snap-frozen in liquid nitrogen, pulverized in pre-cooled sterile mortars, and stored at −80°C. DNA was extracted from homogenized samples, quantified, and analyzed by qPCR targeting the *T. gondii* B1 gene (primers: Table 1) using a two-step amplification protocol (95°C for 2 min; 40 cycles of 95°C for 5 s and 60°C for 30 s). Mouse mActin was used as the internal reference gene (primers: Table 1). Relative parasite burden in each tissue was determined based on normalized TgB1 amplification levels and compared between RH- and Δ*ddx6*-infected groups. Statistical analysis was performed with GraphPad Prism 9.

### Determination of serum IgG antibody and its subtypes in Δddx6 vaccination mice

Serum levels of *T. gondii*‑specific IgG and its subtypes (IgG1, IgG2a) were quantified by indirect enzyme‑linked immunosorbent assay. Briefly, 96‑well microplates were coated with 100 μL per well of soluble tachyzoite antigen (STAg) (10 μg/mL in carbonate buffer, pH 9.6) and incubated at 37°C for 2 h, followed by overnight stabilization at 4°C to ensure optimal antigen immobilization. After coating, plates were blocked with 300 μL of PBST (PBS containing 0.05% Tween‑20) supplemented with 5% skim milk for 2 h at 37°C to minimize non‑specific binding. Wells were then washed five times with PBST (200 μL per wash) and gently tapped dry. Serial dilutions of mouse serum samples were added (100 μL/well), along with negative controls (healthy mouse serum diluted 1:200) and blank controls (diluent only), and incubated at 37°C for 2 h. Following repeated washing, plates were incubated with horseradish peroxidase (HRP)‑conjugated anti‑mouse IgG, IgG1, or IgG2a antibodies (Southern Biotech) (diluted 1:5000 in blocking buffer) for 2 h at 37°C in the dark. After a final wash cycle, 100 μL of TMB substrate was added to each well and developed for 20 min at 37°C in the dark. The reaction was terminated with 50 μL of 2 M H_2_SO_4_, and the optical density at 450 nm was measured using a microplate reader. Data were analyzed with GraphPad Prism 9, and antibody titers were calculated based on a standard curve included in each plate.

### Detection of splenic T lymphocyte subsets in Δddx6 vaccination mice

For immunophenotyping of splenic T cell subsets, single-cell suspensions were first prepared from mouse spleens using mechanical dissociation and filtration through a 70 μm cell strainer, as previously described [28]. The splenocytes were resuspended in flow cytometry staining buffer (PBS containing 1% FBS) and adjusted to a concentration of 1 × 10^7^ cells/mL. For antibody staining, 2 μL of anti-mouse CD3-APC (BioLegend, #100235), 1 μL of CD4-PE (BioLegend, #100407), and 0.5 μL of CD8-PE (BioLegend, #100707) antibody solutions were added to each sample tube containing 100 μL of cell suspension (approximately 1 × 10^6^ cells). The reaction system was gently mixed and incubated at 4°C in the dark for 30 minutes to ensure specific antibody binding while minimizing nonspecific interactions. After incubation, unbound antibodies were removed by centrifuging the samples at 300 ×g for 5 minutes and washing twice with ice-cold PBS. Finally, the cell pellets were resuspended in 500 μL of flow cytometry staining buffer and analyzed using a flow cytometer (CytoFLEX3, China), with appropriate compensation controls and gating strategies to distinguish CD3^+^ CD4^+^ and CD3^+^ CD8^+^ T cell populations. Data were analyzed using FlowJo v10.8.

### Determination of cytokine levels in Δddx6 vaccination mice

Cytokine levels in vaccinated mice were quantified using ELISA. Prior to the assay, reagents were prepared: detection antibody concentrates and HRP-streptavidin (HRP-SA) were stored at 4°C in the dark, while all other components were equilibrated at room temperature for 30 minutes. Required microplate strips were retrieved based on sample numbers, with unused strips sealed in desiccant-containing foil bags and stored at 4°C. For splenocyte processing, mice were aseptically euthanized, and spleens were excised, rinsed with PBS, and mechanically dissociated into single-cell suspensions. After centrifugation at 3500 ×g for 5 minutes at 4°C, red blood cells were lysed using a hypotonic buffer during a 5–10-minute ice bath, and the resulting pellet was resuspended in RPMI-1640 medium supplemented with 10% fetal bovine serum (FBS) for cell counting. Splenocytes were seeded into 24-well plates at 5 × 10^5^ cells per well and stimulated with 10 μg/mL STAg in RPMI-1640/10% FBS. Supernatants were collected at 72 hours (for TNF-α and IL-10 detection) (Proteintech, #KE10002, KE10124) and 96 hours (for IFN-γ detection) (Proteintech, #KE10094). For ELISA, microplates were configured with blank wells (sample diluent only), standard curves, and sample wells. Using a multichannel pipette, 100 μL of standards or samples was added to each well, followed by incubation at 37°C for 2 hours. After washing five times with PBST (0.05% Tween-20), 100 μL of detection antibody was added and incubated for 1 hour at 37°C in the dark. Plates were washed again, incubated with HRP-SA conjugate for 40 minutes, and developed with TMB substrate for 15–20 minutes. The reaction was stopped with 2 M H_2_SO_4_, and optical density was measured at 450 nm (reference: 630 nm). Net OD values were derived by subtracting blank readings, and cytokine concentrations were calculated via four-parameter logistic (4-PL) regression based on the standard curve.

### Mouse protection against acute and chronic infections by Δddx6 vaccination

To assess the vaccine-induced protection, mice were allocated into PBS control and Δ*ddx6* immunization groups (*n* = 10 per group). The Δ*ddx6* tachyzoites were harvested and administered via intraperitoneal injection to the immunized group at a dose of 1 × 10^6^ parasites per mouse, whereas the control group received an equal volume of PBS. A single immunization was performed in all vaccination experiments, and no booster immunization was administered during the study. Thirty days post‑immunization, challenge infection was performed using the highly virulent RH strain (100 tachyzoites per mouse, intraperitoneal injection) or the chronic PRU strain (10 cysts per mouse, oral gavage). For each challenge experiment, freshly harvested parasites from a single preparation were used for all animals. Parasites were enumerated immediately before inoculation, and all mice were challenged on the same day by the same experienced investigator using an identical intraperitoneal injection procedure. These measures were taken to ensure consistency of parasite inoculation and minimize experimental variability among animals. Following infection, mouse survival was monitored daily, and time of death was recorded at 12‑hour intervals. Survival curves were generated using GraphPad Prism version 9 In parallel, brain cyst burdens in PRU‑challenged mice were quantified to evaluate protective efficacy against chronic infection. Briefly, PRU-challenged mice were euthanized at 40 days post-challenge, and whole brains were aseptically collected and homogenized in 2 mL sterile PBS by repeated mechanical disruption. For each mouse, 10 μL of well-mixed brain homogenate was placed on glass slides and examined under light microscopy at 20× magnification. Cysts were identified based on their typical spherical morphology and counted in three technical aliquots per brain by investigators blinded to group allocation. The total number of cysts per brain was calculated according to the dilution factor and total homogenate volume.

### Evaluation of protection against congenital toxoplasmosis by immunization with the Δddx6 strain

Female mice were randomly allocated into control and experimental groups (*n* = 30 per group). The experimental group received intraperitoneal immunization with 1 × 10^6^ tachyzoites of the *T. gondii* Δ*ddx6* strain, while the control group received PBS. Sixty days post-immunization, mice were co-housed with male breeders at a 2:1 female-to-male ratio. Vaginal plugs were checked daily at 7:00, and the day of plug detection was designated as gestational day (GD) 0.5. On GD 8.5, pregnant females were challenged intraperitoneally with 100 wild-type RH strain tachyzoites. On GD 13.5 (5 days post-infection), mice were euthanized, and placental tissues were aseptically collected for further analysis.

### Histological analysis by Hematoxylin and eosin (H&E) staining

Freshly collected mouse tissues were fixed in 4% paraformaldehyde for 24 hours, after which samples were trimmed to expose a smooth surface in a fume hood. Dehydration and paraffin infiltration were performed using an automated tissue processor: tissues were sequentially treated with 75% alcohol (1.5 hours), 85% alcohol (1.5 hours), 90% alcohol (1.5 hours), 95% alcohol (1–1.5 hours), absolute ethanol (two changes, 1–1.5 hours each), alcohol-benzene (5–10 minutes), xylene (two changes, 10–20 minutes each), and finally embedded through three changes of molten paraffin at 65°C (30 minutes, 1 hour, and 1.5 hours, respectively). For embedding, molten paraffin was dispensed into molds, and dehydrated tissues were oriented in the molds, cooled on a −20°C freezing stage, and trimmed into blocks. Sections of 4 μm thickness were cut using a microtome, floated on a 40°C water bath, transferred onto glass slides, and dried at 60°C until paraffin melting was complete. Deparaffinization was conducted with xylene (two changes, 5–10 minutes each), followed by rehydration through a graded ethanol series (absolute ethanol, 95%, 90%, 85%, and 75% alcohol, 2–3 minutes each) and rinsing with distilled water. Sections were stained with hematoxylin for 3–5 minutes, rinsed under tap water, differentiated in 1% acid alcohol for 3–5 seconds, blued in tap water for 5–10 minutes, counterstained with eosin for 1–2 minutes, dehydrated through graded alcohols, cleared in xylene, and mounted with neutral balsam under a coverslip for microscopic examination.

### Collection of tachyzoites and transcriptome sequencing

Tachyzoites of the RH strain and the Δ*ddx6* mutant were harvested for transcriptome sequencing as follows. Briefly, confluent monolayers of HFF cells cultured in T175 flasks were infected with parasites and incubated at 37°C for 48 hours. The medium was then removed, and the cells were gently washed with pre-warmed phosphate-buffered saline (PBS) to eliminate extracellular tachyzoites. The infected HFF cell layer was scraped and mechanically disrupted by passing the cell suspension through a 25-gauge needle 5–8 times to release intracellular parasites. The resulting lysate was filtered through a sterile 4 μm polycarbonate membrane (autoclaved and air-dried) to remove host cell debris. The filtrate was centrifuged at 500 ×g for 10 minutes to pellet the tachyzoites. The pellet was resuspended in TRIzol reagent at a density of 1 × 10^6^ parasites/mL and homogenized by repeated passage through a 25-gauge needle until the solution became clear and transparent. The samples were flash-frozen in liquid nitrogen for 5 minutes and stored at −80°C for long-term preservation. Finally, the samples were shipped on dry ice to Guangzhou GENE DENOVO Biotechnology Corporation for RNA integrity assessment, library construction, and transcriptome sequencing. Three independent biological replicates were performed for each experimental group.

### Immunofluorescence assay

HFF cells were seeded onto ethanol-sterilized coverslips placed in 24-well plates. After 24 h of culture, cells were infected with freshly egressed tachyzoites of RH and Δ*ddx6* strains for 1–2 h, followed by three PBS washes to remove non-invaded parasites. Infected monolayers were fixed with 4% paraformaldehyde for 30 min at room temperature, permeabilized with 0.2% Triton X-100 for 30 min, and blocked with 5% BSA for 1 h. Cells were then incubated overnight at 4°C with primary antibodies against specific organelle markers: mouse anti-ACP, mouse anti-BiP, rabbit anti-STX6, rabbit anti-Tubulin, rabbit anti-ROP5, rabbit anti-Cen1, together with rabbit anti-GAP45 and mouse anti-IMC1 to label the parasite membrane and IMC. After three PBS washes, samples were incubated for 1 h at room temperature with appropriate secondary antibodies: Alexa Fluor 488-conjugated goat anti-mouse IgG and Alexa Fluor 594-conjugated goat anti-rabbit IgG; or Alexa Fluor 594-conjugated goat anti-mouse IgG and Alexa Fluor 488-conjugated goat anti-rabbit IgG. Nuclei were stained with DAPI for 5 min, followed by three PBS washes. Coverslips were mounted on glass slides using anti-fade mounting medium and sealed with nail polish. Images were acquired using a Nikon A1 laser scanning confocal microscope and processed with NIS-Elements software.

### Statistical analysis

All of the statistical analyses were performed using the GraphPad Prism 9 software. All measurement data were expressed as mean ± standard deviation (X ± SD), and 95% confidence intervals were calculated for major outcome measurements. Paired comparisons between groups were performed using t-tests, and comparisons among multiple groups were conducted using one-way ANOVA and two-way ANOVA according to the experimental design. To ensure the reliability and reproducibility of the experimental results, all experiments were independently repeated three times. Statistical significance was defined as *p* < 0.05 (*p < 0.05, **p < 0.01, ***p < 0.001, ****p < 0.0001). Effect sizes and 95% confidence intervals were reported where appropriate for major outcome measurements.

## Results

### Successful construction of the T. gondii Δddx6 strain using CRISPR/Cas9

**Figure 1.**
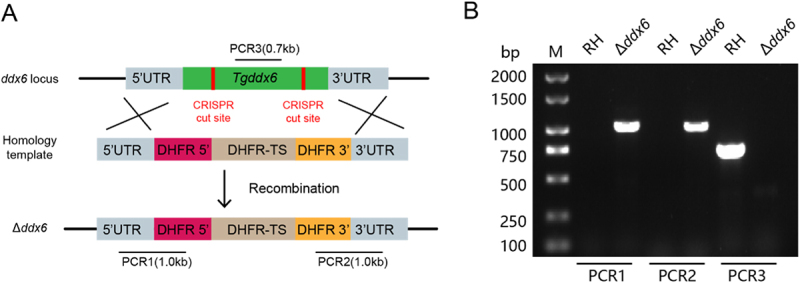
Construction and identification of the Δ*ddx6* gene-deleted strain.

The *ddx6* gene was successfully disrupted in the virulent *T. gondii* RH strain (Dku80) using a CRISPR/Cas9-mediated homologous replacement strategy. The entire coding sequence (CDS) of *ddx6* was replaced with a pyrimethamine-resistant DHFR-TS cassette to ensure complete knockout (Figure 1(A)). Successful gene knockout was confirmed by diagnostic PCR analysis using three specific primer sets (PCR1, PCR2, PCR3) designed to amplify regions spanning the integration sites and the deleted CDS (Figure 1(B)). PCR1 and PCR2 yielded the expected larger fragments of 1036 bp and 1006 bp, respectively, in the Δ*ddx6* clone compared to the wildtype (RH) control, confirming the successful integration of the DHFR-TS cassette. Conversely, PCR3, which amplifies a 714 bp fragment within the deleted CDS region, produced a band only in the wildtype parasites, verifying the absence of the target gene in the knockout strain. These molecular analyses comprehensively verify the successful generation of a TgDDX6-deficient *T. gondii* RH strain, designated as Δ*ddx6*.

(A) Schematic diagram of the construction of the Δ*ddx6* strain. (B) Nucleic acid-level identification of Δ*ddx6* monoclonal deletion strains. M: 2000bp Marker; PCR1: 1036 bp; PCR2: 1006 bp; PCR3: 714 bp.

### The Δddx6 strain is moderately impaired in vitro while attenuated in vivo

**Figure 2.**
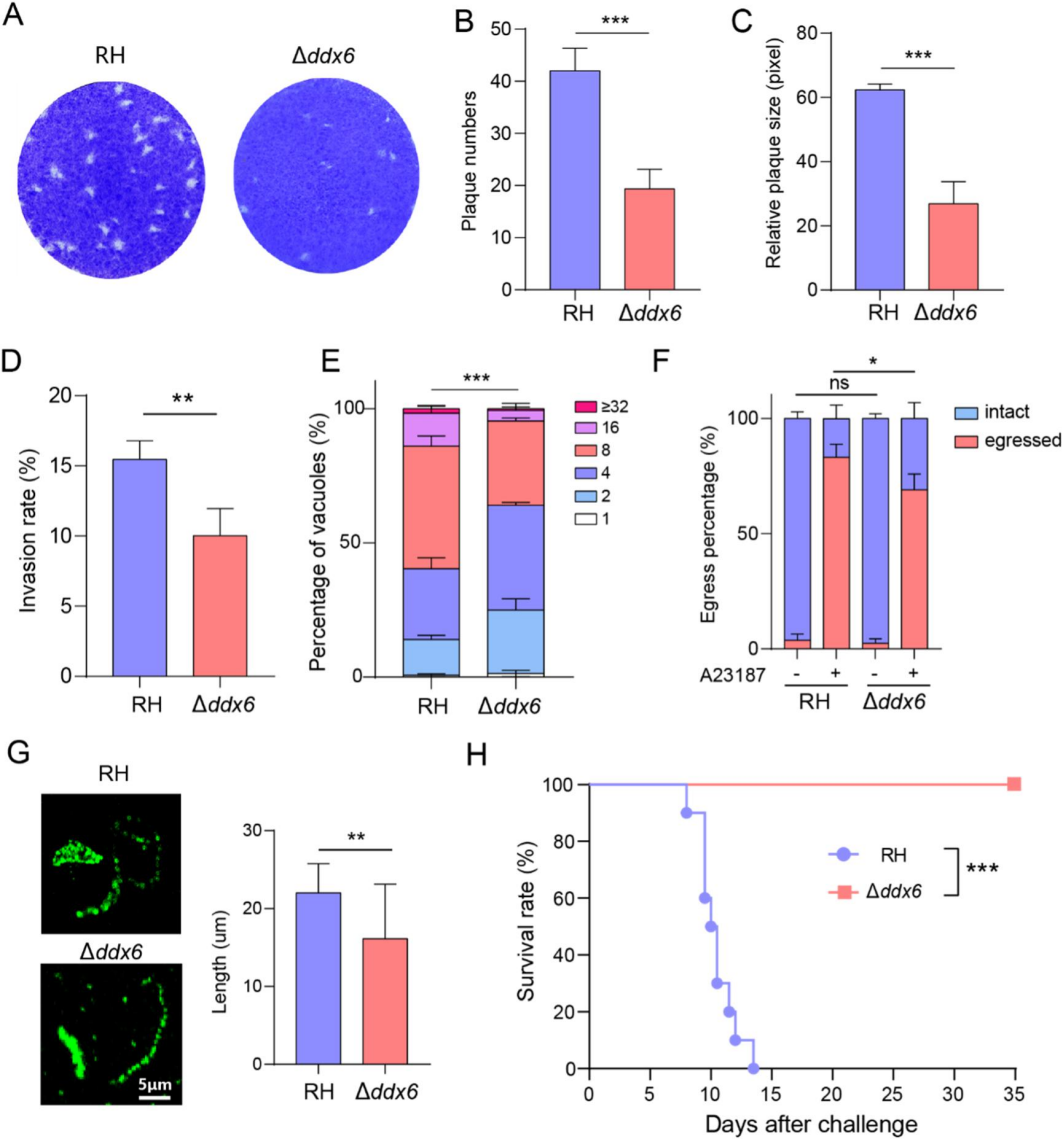
Phenotypic changes of the Δ*ddx6* gene-deleted strain.

To systematically evaluate the phenotypic consequences of DDX6 depletion, we first assessed the lytic cycle capabilities of the Δ*ddx6* strain *in vitro*. Plaque assay revealed that the Δ*ddx6* strain formed fewer and smaller plaques compared to RH strain (Figure 2(A-C)), indicating a defect in cell invasion, replication, or subsequent spreading to neighboring cells. To dissect the specific stages affected, we performed detailed functional assays. The invasion assay showed that the average invasion rate of Δ*ddx6* tachyzoites was significantly reduced to 10.02% ± 1.94%, compared to 15.46% ± 1.33% for the RH strain (Figure 2(D)). Subsequently, the intracellular replication of the Δ*ddx6* strain was also significantly impaired, as evidenced by a notable decrease in the number of tachyzoites per PVs (Figure 2(E)). Furthermore, the egress efficiency of the Δ*ddx6* strain was also compromised, with an average egress rate of 69% ± 6.84%, which was significantly lower than the 83.11% ± 5.65% observed in the RH strain (Figure 2(F)). The gliding motility assay confirmed another key defect, as the average trail length of Δ*ddx6* tachyzoites (16.13 ± 7 μm) was considerably shorter than that of RH tachyzoites (22 ± 3.77 μm) (Figure 2(G)). We next evaluated the virulence of the Δ*ddx6* strain in a murine model. Mice infected with the RH strain succumbed to acute toxoplasmosis within 14 days post-infection (Figure 2(H)). In contrast, all mice infected with the Δ*ddx6* strain survived the observation period, demonstrating severe attenuation of virulence.

(A) Crystal violet staining of Δ*ddx6* plaques. (B) Statistical graph of the number of Δ*ddx6*plaques (*n* = 3). (C) Statistical graph of the area of Δ*ddx6* plaques (*n* = 3). (D) Statistical graph of the Δ*ddx6* invasion assay (compared with the wild-type RH strain group (*n* = 3). (E) Statistical graph of the Δ*ddx6* proliferation assay compared with the wild-type RH strain group (*n* = 3). (F) Statistical graph of the Δ*ddx6* proliferation assay. (G) Gliding trails of Δ*ddx6* and statistical graph of gliding trail length (*n* = 20). (H) Statistical graph of the Δ*ddx6* mouse virulence assay (compared with the RH strain group, *n* = 10). **: *p* < 0.005, ***: *p* < 0.001.

### The Δddx6 strain is severely attenuated in vivo and triggers a humoral immune response

**Figure 3.**
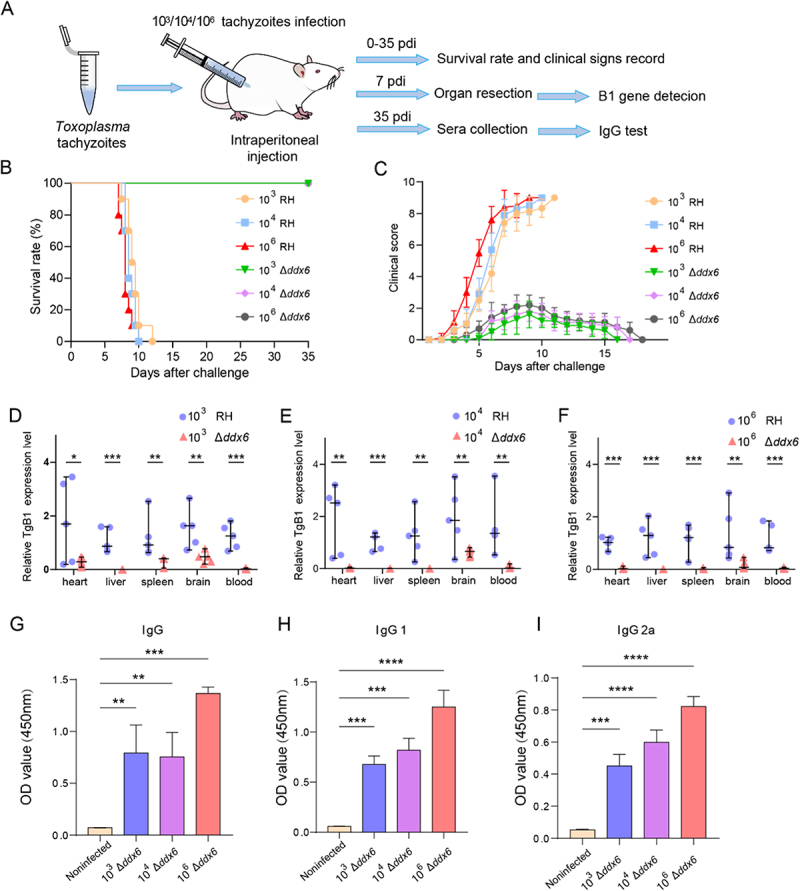
Virulence evaluation of Δ*ddx6* in mice.

To further evaluate the virulence of the Δ*ddx6* strain *in vivo*, ICR mice were infected intraperitoneally with different doses (10^3^, 10^4^, and 10^6^ tachyzoites) of either the wildtype or Δ*ddx6* tachyzoites, and survival rates were monitored for 35 days (Figure 3(A)). All mice infected with the RH strain succumbed to acute toxoplasmosis within 12 days post-infection, regardless of the challenge dose. In stark contrast, all mice infected with the Δ*ddx6* strain survived throughout the entire observation period at all challenge doses, demonstrating severely attenuation of virulence (Figure 3(B)). Clinical scores remained significantly lower in Δ *ddx6*-infected mice compared to RH-infected controls, with all signs resolving completely by day 21 (Figure 3(C)).

(A) Schematic diagram of the *in vivo* virulence evaluation experimental procedure. (B) Statistical graph of the survival rate of mice infected with different doses of *T. gondii* (*n* = 10). (C) Clinical sign scores of mice infected with different doses of *T. gondii*; clinical scores range from 0 (no signs) to 10 (all signs) (*n* = 10). (D-F) Parasite burden in the heart, liver, spleen, brain and blood of mice infected with 1 × 10^3^, 1 × 10^4^, 1 × 10^6^ tachyzoites (*n* = 4–5). (G-I) Statistical graph of IgG, IgG1, IgG2a changes in mice infected with different doses of *T. gondii* (*n* = 3); statistical analysis was performed using unpaired t-test. Bars = mean ± standard error of the mean (SD). *: *p* < 0.05, **: *p* < 0.005, ***: *p* < 0.001, ****: *p* < 0.0001.

To delineate the parasitic dynamics underlying the stark contrast in survival, we quantified parasite burdens in key tissues (heart, liver, spleen, brain, and peripheral blood) during the acute phase of infection (7 dpi). Quantitative PCR analysis targeting the TgB1 gene revealed substantially different kinetics between the RH and Δ*ddx6* strains. While RH parasites rapidly proliferated and disseminated, establishing high parasite loads in all examined tissues at all doses, the Δ*ddx6* strain exhibited significantly lower parasite loads across every tissue type and at every challenge dose (Figure 3(D-F)). This marked and consistent reduction in parasitic burden provides a clear pathological basis for the complete absence of mortality observed in Δ*ddx6* infected animals.

The immunogenicity of the attenuated strain was further assessed by measuring the humoral immune response at 35 dpi. Infection with the Δ*ddx6* strain elicited a robust and durable antigen-specific antibody response. Notably, mice infected with the 10^6^ dose mounted a substantially stronger and more persistent humoral immune response compared to those receiving lower doses (Figure 3(G-I)). Based on this optimal immunogenicity profile, the 10^6^ dose was selected for all subsequent vaccination and challenge experiments to evaluate protective efficacy.

### Vaccination with Δddx6 tachyzoites elicits robust and durable immune responses

**Figure 4.**
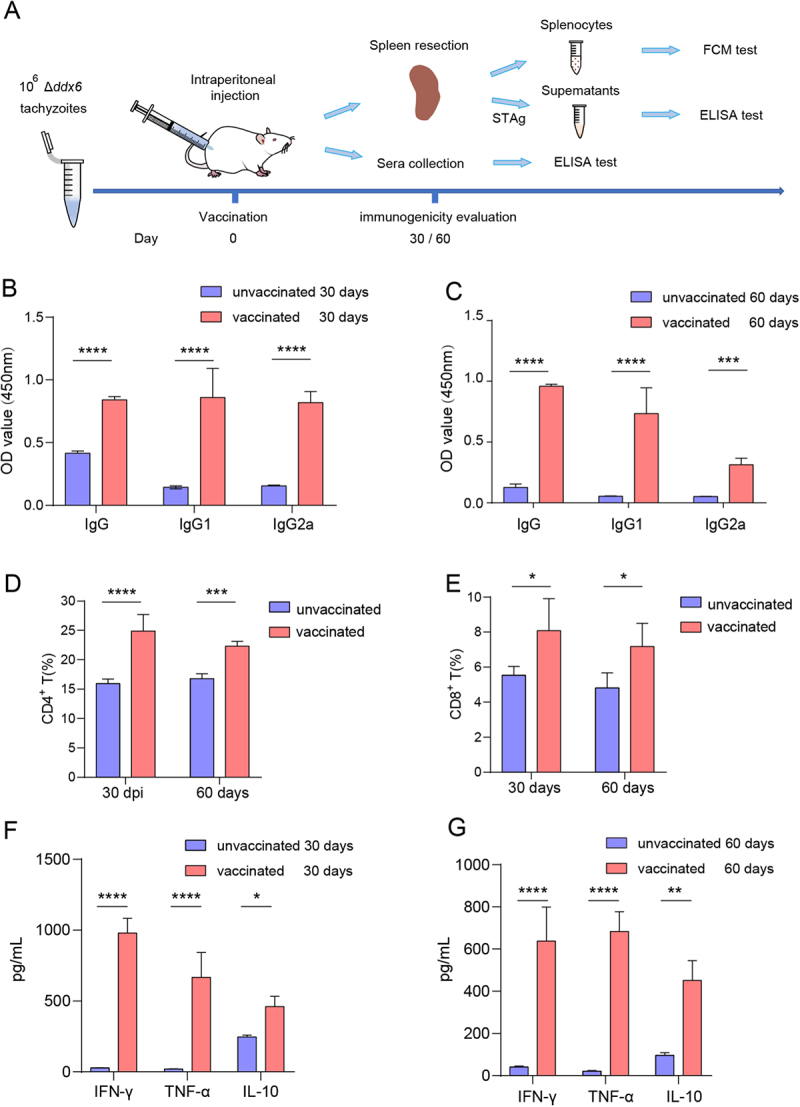
Immunogenicity analysis of mice infected with Δ*ddx6*.

To evaluate the immunogenicity of the attenuated Δ*ddx6* strain as a potential live-attenuated vaccine, ICR mice were immunized intraperitoneally with 10^6^ tachyzoites, and immune responses were assessed at 30- and 60-days post-vaccination (dpv) (Figure 4(A)). Analysis of the humoral immune response demonstrated that vaccination with the Δ*ddx6* strain elicited a strong antigen-specific antibody response. At 30 dpv, immunized mice exhibited significantly elevated levels of *T. gondii*-specific total IgG, as well as IgG1 and IgG2a subclasses, compared to unvaccinated controls (Figure 4(B)). Remarkably, this robust antibody response persisted through 60 dpv, with vaccinated mice maintaining significantly higher levels of all antibody isotypes compared to controls (Figure 4(C)), demonstrating the durability of the humoral immunity induced by the Δ*ddx6* vaccination.

(A) Schematic diagram of the immunogenicity analysis experimental procedure. (B) Statistical graph of IgG changes in mice 30 days and 60 days (C) after immunization (*n* = 3); (D) Statistical graph of the proportion of CD4^+^T cells and CD8^+^T cells (E) in mouse spleens 30 days and 60 days after immunization (*n* = 5). F Splenocytes were isolated from unvaccinated or Δ*ddx6*-vaccinated mice at 30 days and 60 days (G) post-immunization and stimulated with STAg. Levels of IFN-γ, TNF-α, and IL-10 in the culture supernatants were quantified by ELISA. Data are presented as optical density (OD) values measured at 450 nm and represent the mean ± SD from three independent experiments. *: *p* < 0.05, **: *p* < 0.005, ***: *p* < 0.001, ****: *p* < 0.0001.

The cellular immune response was similarly robust and sustained. Flow cytometric analysis of splenocytes revealed significantly increased proportions of both CD4^+^ T cells (Figure 4(D)) and CD8^+^ T cells (Figure 4(E)) in immunized mice compared to controls at both 30 and 60 dpv. To evaluate antigen-specific T cell function, splenocytes isolated at 30 and 60 dpv were stimulated with soluble tachyzoite antigen (STAg) and cytokine production was measured by ELISA. Consistent with T cell activation, cytokine analysis showed substantially elevated levels of key Th1-type cytokines in immunized mice, including IFN-γ and TNF-α (Figure 4(F,G)). Additionally, the immunoregulatory cytokine IL-10 was also significantly increased in vaccinated animals (Figure 4(F,G)), suggesting the induction of a modulated immune response. Importantly, these enhanced cytokine responses persisted at 60 dpi, indicating the durability of the cellular immunity induced by Δ*ddx6* vaccination.

### Vaccination with Δddx6 confers potent protection against acute and chronic toxoplasmosis

**Figure 5.**
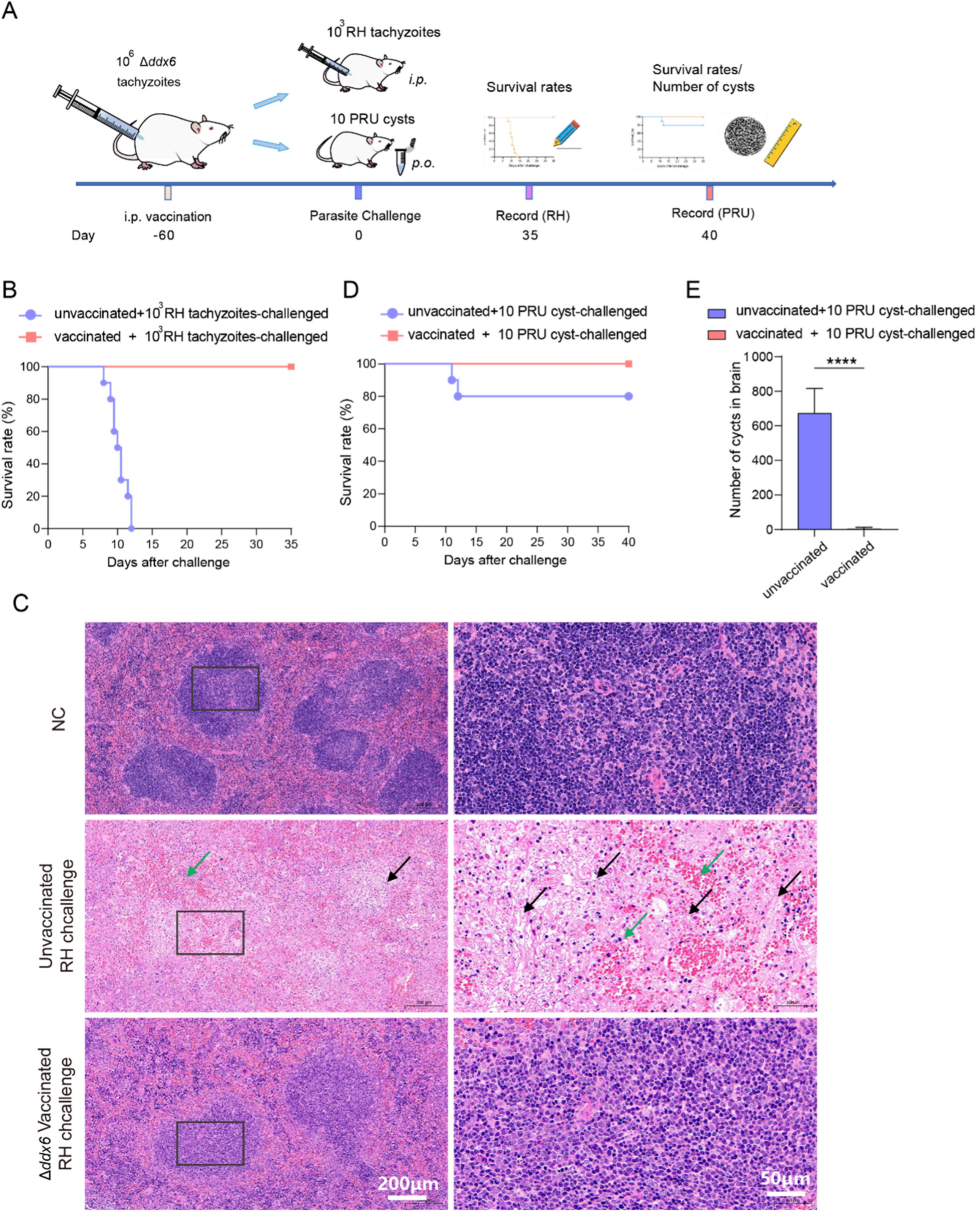
Δ*ddx6* immunization confers dual protection against acute and chronic toxoplasmosis in mice.

To evaluate the protective efficacy of the Δ*ddx6* strain, mice were immunized intraperitoneally with 10^6^ tachyzoites and subsequently challenged with either highly virulent RH tachyzoites or the cyst-forming PRU (type II) strain at 60 days post-immunization (Figure 5(A)).

(A) Schematic diagram of the study design. (B) Survival rates of mice following acute infection with the RH strain (*n* = 10). (C) Histopathological analysis of splenic tissues (H&E staining). Left column: 200 μm field; right column: 50 μm magnified view of the black square. NC group: White pulp and red pulp are well-demarcated; white pulp consists of periarteriolar lymphoid sheaths and lymphoid follicles with clear structure; red pulp is uniformly distributed without significant inflammatory infiltration. Unvaccinated RH challenge group: Disorganized splenic structure with extensive necrosis (black arrows), nuclear fragmentation or lysis, cytoplasmic disintegration, and multifocal hemorrhages (green arrows); residual lymphocytes are markedly reduced and disorderly arranged. Vaccinated RH challenge group: Architecture is well preserved, similar to the NC group. (D) Survival rate of mice with chronic *T. gondii* PRU stain infection (*n* = 10). (E) Number of cysts in the brains of mice with chronic*T. gondii*infection (*n* = 8/10). Statistical analysis was performed using unpaired t-test. *: *p* < 0.05, **: *p* < 0.005, ***: *p* < 0.001, ****:*p* < 0.0001.

In the acute infection model, when challenged with 10^3^ RH tachyzoites, all unvaccinated control mice succumbed to acute toxoplasmosis within 12 days post-infection. In stark contrast, 100% of Δ*ddx6*-vaccinated mice survived the challenge, demonstrating complete protection against the virulent RH strain (Figure 5(B)). Histopathological examination of spleen tissues at 7 days after RH challenge provided further evidence of protection. In the NC group and Δ*ddx6*-vaccinated group, splenic architecture remained well-preserved, with clear demarcation between white pulp (composed of periarteriolar lymphoid sheaths and lymphoid follicles) and red pulp. The red pulp showed uniform distribution without significant inflammatory cell infiltration. In stark contrast, the unvaccinated group exhibited severe splenic structural disruption, characterized by extensive coagulative necrosis accompanied by multifocal hemorrhages (Figure 5(C)).

The protective efficacy of Δ*ddx6* vaccination was further validated in the chronic infection model. Following oral challenge with 10 PRU cysts, unvaccinated control mice exhibited progressive disease development with a final survival rate of 80% by 35 days post-infection. Δ*ddx6*-vaccinated mice demonstrated complete protection with 100% survival throughout the observation period (Figure 5(D)). Notably, vaccination conferred nearly sterilizing immunity against chronic infection. Quantitative analysis of brain cyst burdens at 40 days post-challenge revealed a profound reduction in parasite persistence. Unvaccinated controls developed substantial cyst loads (672 ± 50.91), whereas in the vaccinated group, brain cysts were detected in only 1 out of 10 vaccinated mice (3.3 ± 3.3), representing a 99.5% reduction in parasite burden (Figure 5(E)).

In summary, Δ*ddx6* vaccination confers robust protection, not only ensuring 100% survival upon acute lethal challenge but also inducing a state of near-sterilizing immunity against chronic infection.

### Vaccination with Δddx6 confers protection against congenital toxoplasmosis

**Figure 6.**
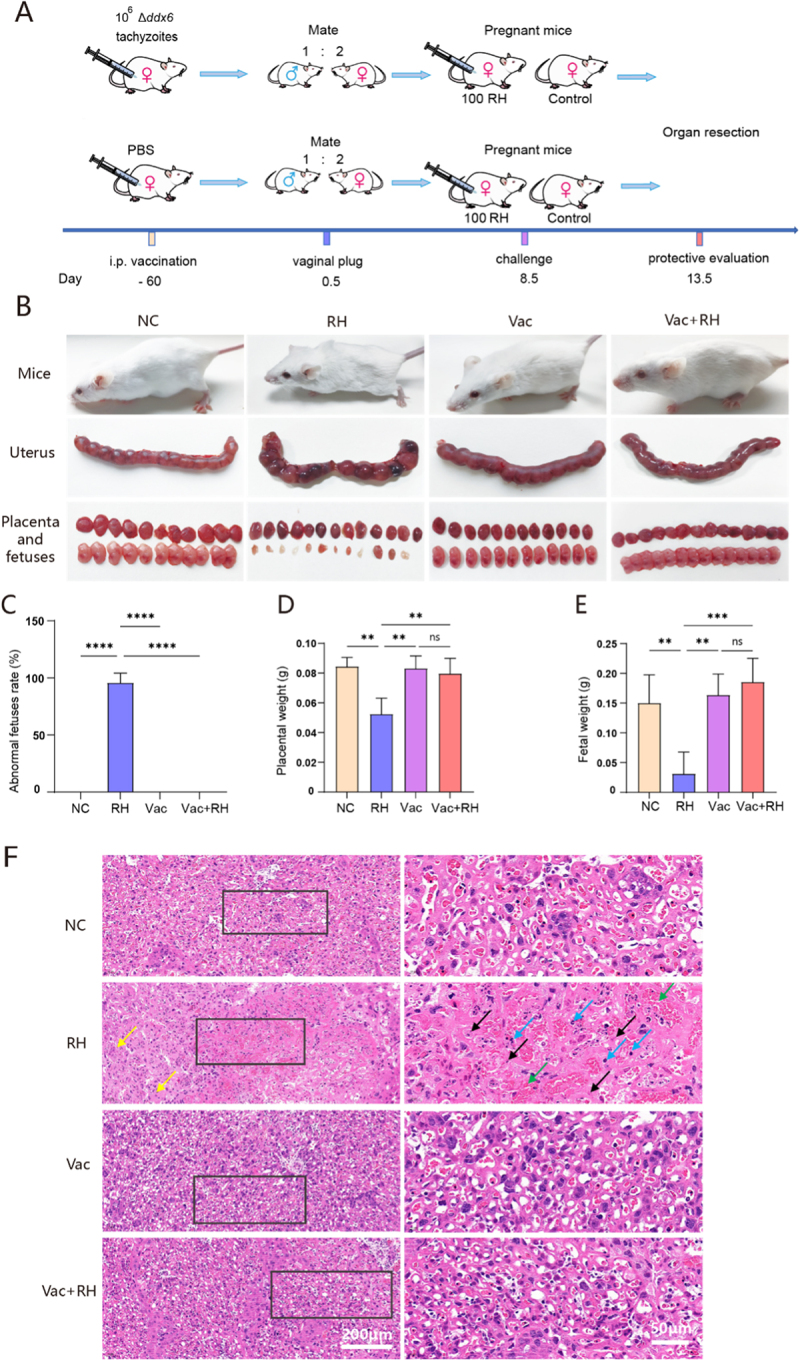
Δ*ddx6* immunization protects against congenital toxoplasmosis in a pregnancy model.

To evaluate the protective efficacy of Δ*ddx6* vaccination against vertical transmission, we established a pregnant mouse model of congenital toxoplasmosis. Female mice were vaccinated intraperitoneally with 10^6^ Δ*ddx6* tachyzoites or served as controls, followed by mating and challenge with 10^2^ RH tachyzoites at 8.5 days of gestation (GD) (Figure 6(A)).

(A) Schematic diagram illustrating the experimental timeline of vaccination, mating, and challenge in the pregnancy model. (B) Representative phenotypic observations of dams, uteri, placentas, and fetuses from each group post-infection. Vac+RH dams maintained normal uterine architecture with well-developed fetuses, while RH-challenged controls showed severe uterine hemorrhage, placental shrinkage, and fetal resorption (*n* = 4). (C) Abortion rates in infected pregnant mice. Vac+RH significantly reduced fetal loss compared to the challenged control group (*n* = 4). (D) Placental weights across experimental groups (*n* = 4). Vac+RH preserved placental mass, indicating maintained placental function despite infection. (E) Fetal weights in each group (*n* = 4). Fetuses from Vac+RH dams showed normal development compared to the growth-restricted fetuses in challenged controls. (F) Histopathological analysis of placental tissues from different experimental groups (H&E staining). The placental labyrinth zone in the RH-challenged control group shows severe structural disorganization with patchy necrosis (black arrows), characterized by nuclear fragmentation and deposition of fibrin-like material. The necrotic areas appear loosely structured with mild peripheral hemorrhage (green arrows) and scattered infiltration of granulocytes and lymphocytes (blue arrows). The remaining villi are markedly thickened (yellow arrows) with obscured structures, and maternal blood sinuses contain reduced blood volume. Black boxes indicate the areas selected for higher-magnification views (50 μm scale). In contrast, the NC, Vac, and Vac+RH groups display normal placental architecture: the labyrinth zone consists of well-organized maternal sinuses and villi, each composed of three layers of trophoblasts surrounding fetal blood vessels and mesenchymal tissue. Fetal vessels contain nucleated red blood cells, and no significant necrosis or inflammatory infiltration is observed. Statistical analysis was performed using unpaired t-test. **: *p* < 0.005, ***: *p* < 0.001, ****: *p* < 0.0001.

Gross examination revealed striking differences among the groups (Figure 6(B)). The NC (naïve control) group showed normal uterine architecture with well-developed fetuses, while the RH challenge control group exhibited severe pathological changes including uterine hemorrhage and resorption sites. In contrast, the Vac+RH (vaccinated and RH strain challenged) group maintained relatively normal uterine structure with preserved fetal development, similar to the Vac group.

Quantitative analysis demonstrated significant protection in vaccinated mice. The RH challenge control group showed a high percentage of abnormal fetuses (95.83 ± 4.18%), whereas the Vac+RH group exhibited complete protection with no observed fetal resorption or developmental abnormalities (Figure 6(C)). Placental weight analysis revealed complete protection in vaccinated groups (Figure 6(D)). While RH challenge substantially reduced placental weight (0.053 ± 0.005 g) compared to NC controls (0.085 ± 0.003 g), both the Vac group (0.083 ± 0.004 g) and Vac+RH group (0.080 ± 0.005 g) maintained placental weights comparable to normal levels, demonstrating effective preservation of placental function. Similarly, fetal weight was better preserved in the Vac+RH group (0.186 ± 0.02 g) compared to the RH challenge group (0.031 ± 0.018 g) (Figure 6(E)).

qPCR analysis of placental tissues further revealed upregulated FOXP3 expression and downregulated RORγt expression in the Vac+RH group, suggesting a shift toward immunoregulatory balance at the maternal-fetal interface (Figure S2). Histopathological examination of placental tissues provided further evidence of protection (Figure 6(F)). Compared to the normal placental architecture observed in the NC group, the RH challenge group exhibited severe structural disruption. This included extensive necrosis, fibrin deposition, hemorrhage, and inflammatory infiltration. Notably, both the Vac group and the Vac+RH group maintained placental integrity comparable to the NC group, with preserved tissue structure and absence of significant pathological changes. These results demonstrate that Δ*ddx6* vaccination effectively protects against infection-induced placental damage, maintaining normal histological architecture despite pathogen challenge. These results demonstrate that Δ*ddx6* vaccination effectively protects against infection-induced placental damage, maintaining normal histological architecture despite pathogen challenge.

### Transcriptomic profiling reveals widespread gene expression dysregulation in the Δddx6 strain

To gain initial insights into the molecular basis of the attenuation phenotype observed in the Δ*ddx6* strain, we performed a comparative transcriptome analysis between the mutant and wild-type RH tachyzoites. Given that TgDDX6 encodes a conserved DEAD-box RNA helicase known to be involved in post-transcriptional gene regulation, such as mRNA stability and translation [29] (Figure S1), we hypothesized that its deletion would lead to broad alterations in gene expression, potentially explaining the observed defects in invasion, replication, and virulence.

**Figure 7.**
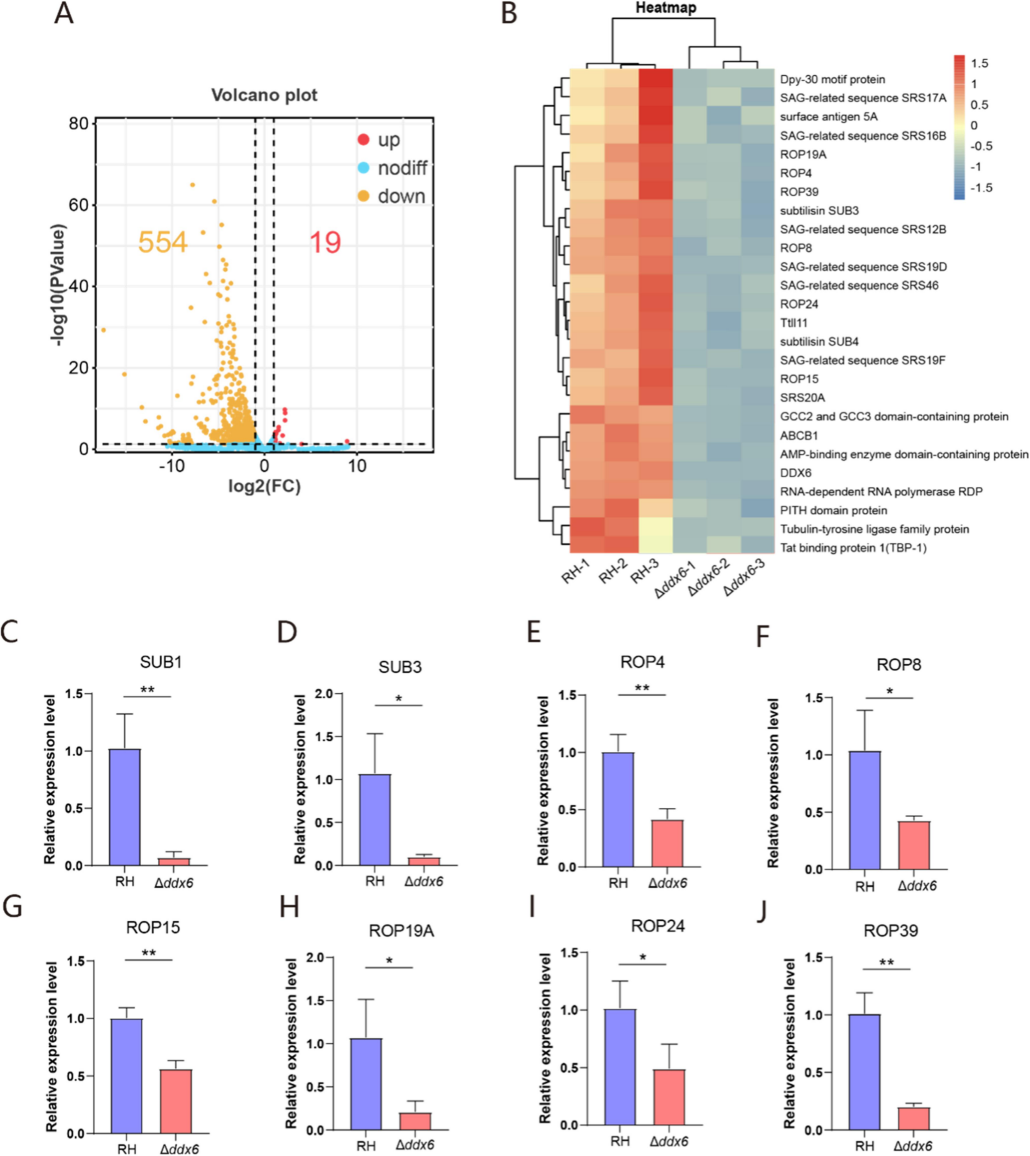
Mechanism analysis of the effect of Δ*ddx6* on the growth and development of *T. gondii*.

RNA sequencing revealed substantial transcriptomic reprogramming in the Δ*ddx6* strain. Differential expression analysis identified 573 significantly dysregulated genes (fold-change > 2, adjusted *p*-value < 0.05), with a striking imbalance of 554 genes downregulated and only 19 genes upregulated in the mutant (Figure 7(A)). This pronounced 29:1 ratio of down-regulated to up-regulated genes suggested that TgDDX6 may primarily function as a positive regulator of gene expression in *T. gondii*. Unsupervised hierarchical clustering of these differentially expressed genes showed clear segregation between the wild-type and Δ*ddx6* transcriptomes, indicating fundamental alterations in the global gene expression landscape following DDX6 deletion (Figure 7(B)).

(A) Volcano plot of differentially expressed genes (DEGs) of Δ*ddx6*. X-axis: log_2_-transformed fold change of gene expression; Y-axis: statistical significance of differences in gene expression changes. (B) Heatmap of partial DEGs of Δ*ddx6*; the redder the color, the higher the gene expression level. (C-J) RT-qPCR detection of the mRNA levels of Δ*ddx6* DEGs, showing the relative expression levels of SUB1, SUB3, ROP4, ROP8, ROP15, ROP19A, ROP24, and ROP39 genes in the RH group and Δ*ddx6* group; *p < 0.05, **p < 0.01.

KEGG pathway enrichment analysis showed that the dysregulated genes were significantly enriched in three major categories (Figure S3(A)). Metabolic pathways constituted the largest group, including carbohydrate, amino acid, and nucleotide metabolism. Organismal system pathways, particularly those involved in environmental adaptation and signal transduction, were also prominently represented. Additionally, signal transduction pathways mediating cellular responses to external stimuli showed significant enrichment. These findings suggest that DDX6 deficiency broadly impacts metabolic homeostasis and signal transduction processes essential for parasite virulence.

GO (Gene Ontology) analysis further clarified the functional consequences of DDX6 deletion across three categories (Figure S3(B)). For biological processes, the most significantly enriched terms were cellular processes and metabolic processes. Within molecular functions, catalytic activity constituted the predominant category. Cellular component analysis revealed strong enrichment for cellular anatomical entities, indicating substantial impacts on cellular organization and structure.

Notably, among the downregulated genes were critical virulence factors essential for host cell invasion and parasite survival. These included rhoptry proteins (ROPs) that mediate host cell penetration and PV formation, subtilisin-like proteases (SUB) involved in parasite maturation and invasion, and surface antigens (SAGs) critical for host cell attachment and immune evasion. To validate the RNA-seq results, we performed qPCR analysis on key virulence-associated genes, which confirmed significant downregulation of SUB1, SUB3, ROP4, ROP 8, ROP1, ROP 19A, ROP 24, and ROP39 in the Δ*ddx6* strain compared to RH parasites (Figure 7(C-J)).

**Figure 8.**
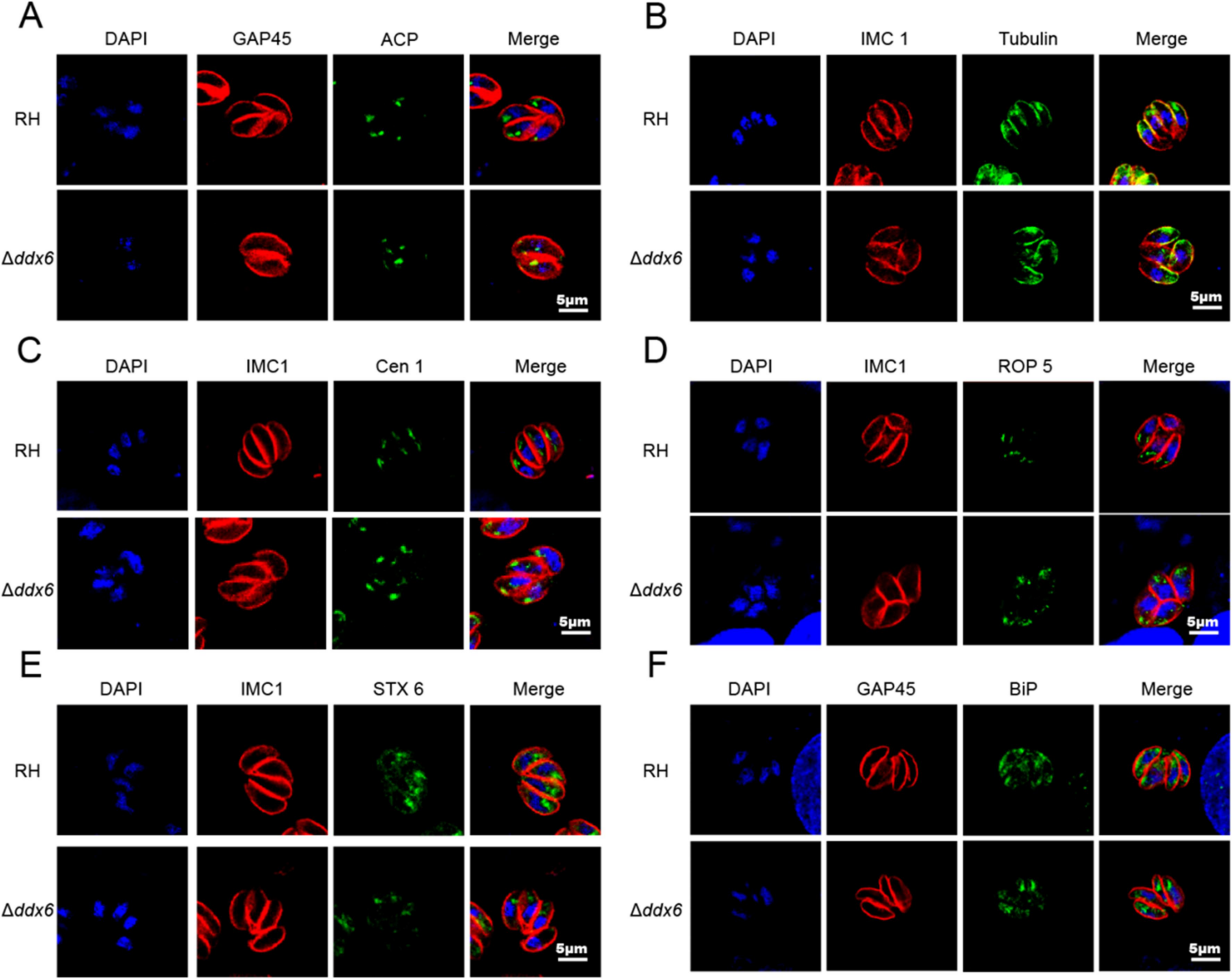
Immunofluorescence analysis of organelle morphology in tachyzoites.

Further, KEGG and GO analyses also revealed significant enrichment of terms related to intracellular transport and cellular processes among downregulated genes, including those encoding proteins critical for vesicular trafficking and organelle maintenance. We therefore examined whether these transcriptional changes might affect organelle morphology and function (Figure 8(A-F)). Immunofluorescence microscopy using organelle-specific markers revealed that while most organelles—including the apicoplast (ACP), microtubules (Tubulin), centrosome (Cen 1), rhoptries (ROP 5), and endoplasmic reticulum (BiP)—maintained normal morphology in Δ*ddx6* parasites, the trans-Golgi network (marked by STX6) exhibited pronounced fragmentation and reduced size compared to wild-type parasites (Figure 8(E)). This organelle-specific structural defect may be associated with the attenuated virulence phenotype of the Δ*ddx6* strain by potentially disrupting proper protein processing and trafficking essential for parasite pathogenesis.

Representative confocal microscopy images showing the subcellular localization of major organelles in wild-type RH and Δ*ddx6* tachyzoites. Organelles were labeled with specific markers: (A) Apicoplast (ACP), (B) Microtubules (Tubulin), (C) Centrosome (Cen1), (D) Rhoptries (ROP5), (E) Trans-Golgi network (STX6) and (F) Endoplasmic reticulum (BiP). Nuclei are stained with DAPI (blue). Scale bars = 5 μm.

Collectively, this transcriptomic profiling provides important molecular clues, suggesting that TgDDX6 may play a critical role in the regulatory network associated with *T. gondii* pathogenicity.

## Discussion

The development of a safe and effective vaccine remains a paramount goal in the fight against toxoplasmosis. In this study, we constructed a live-attenuated vaccine candidate by disrupting the *ddx6* gene, a member of the DEAD-box RNA helicase family, in the virulent RH strain of *T. gondii*. Our comprehensive evaluation demonstrates that the Δ*ddx6* strain exhibits a unique combination of profound attenuation and potent immunogenicity, conferring sterilizing immunity against acute, chronic, and congenital toxoplasmosis in murine models. This work not only presents a promising vaccine candidate but also provides new insights into the role of RNA helicases in apicomplexan parasite biology.

The most striking feature of the Δ*ddx6* strain is its severely attenuated phenotype. Unlike some genetically engineered strains that may only exhibit partial attenuation or residual virulence at high doses [18], the Δ*ddx6* strain was completely avirulent even at a challenge dose of 10^6^ tachyzoites. This profound attenuation is particularly noteworthy when compared to strategies that require the disruption of multiple genes to achieve a similar safety profile. For instance, the attenuation of the *T. gondii* RH strain was achieved through double gene knockout, targeting *ompdc* and *uprt* [30], while protective immunity against both acute and chronic toxoplasmosis was elicited by a mutant deficient in lactate fermentation, which also involved the knockout of two genes [31]. Notably, even with single-gene deletion strategies, the level of protection can vary significantly. For example, the live-attenuated PruΔ*gra72* strain, while eliciting a strong Th1-biased immune response and conferring robust protection against less virulent strains, only provided partial protection against acute infection by the highly virulent Type I RH tachyzoites [16]. Similarly, immunization with the RH Δ*had2a* strain, another single-gene knockout mutant, conferred only partial protective immunity against acute infection with highly virulent tachyzoites [32]. The fact that disruption of the single DDX6 gene in our study resulted in a level of attenuation comparable to these double-gene knockout mutants underscores the pivotal role of DDX6 as a high-level regulatory node. The rapid clearance of the Δ*ddx6* strain from host tissues significantly mitigates the risk of vaccine strain persistence or reversion to virulence, a critical safety concern for live-attenuated vaccines [33]. This suggests that targeting global regulators like DDX6, which sit atop regulatory hierarchies, may yield a more genetically stable and safer attenuated phenotype compared to targeting single virulence factors or even multiple metabolic genes.

Although the Δ*ddx6* strain exhibited functional impairments in key stages of its life cycle—including reduced invasion efficiency, slowed intracellular replication, and compromised gliding motility during *in vitro* culture—these defects did not significantly impact its immunogenicity in mice. Notably, the Δ*ddx6* strain could be stably expanded via *in vitro* cell culture, which is essential for vaccine production. This cultivability indicates that while DDX6 deletion affects multiple pathogenic processes in *T. gondii*, it does not completely disrupt the parasite’s fundamental metabolism or proliferative capacity, thereby supporting the feasibility of large-scale production of this live-attenuated vaccine candidate.

In terms of immune response, vaccination with the Δ*ddx6* strain elicited a robust Th2-type immune response in mice, characterized by high levels of antigen-specific IgG antibodies, activated CD4^+^ and CD8^+^ T cells, and key effector cytokines such as IFN-γ and TNF-α. This balanced and potent immune response is crucial for effective control of *T. gondii* infection. The ability of the Δ*ddx6* strain to induce such comprehensive protective immunity may be attributed to its nature as a live vaccine presenting a broad antigenic repertoire, coupled with the gene expression reprogramming resulting from DDX6 deletion, which may alter the presentation pattern or intensity of parasite antigens, thereby optimizing host immune recognition.

The protective efficacy of the Δ*ddx6* vaccine was rigorously evaluated across the principal clinical forms of toxoplasmosis. In the acute infection model, immunization conferred 100% survival against a lethal challenge with the highly virulent RH strain Histopathological examination confirmed the absence of significant tissue damage in vaccinated mice, underscoring the vaccine’s ability to prevent acute pathology. Notably, the vaccine induced near-sterilizing immunity against chronic infection, achieving a 99.5% reduction in brain cyst burden following challenge with the type II PRU strain. This dramatic decrease in cyst formation is a critical advance, as preventing the establishment of chronic infection has been a major hurdle in toxoplasmosis vaccine development [12,34]. While post-challenge assessment of humoral and cellular immune responses was not conducted in this study, the pre-challenge immune data—including robust *T. gondii*-specific IgG levels, IgG subclass responses, and CD4^+^/CD8^+^ T cell activation—together with the observed protection, strongly support the efficacy of the Δ*ddx6* vaccine. Future studies will examine post-challenge immune dynamics to further elucidate the mechanisms underlying the observed protection.

Most significantly, the Δ*ddx6* vaccine demonstrated complete protection against vertical transmission in a congenital model. Vaccinated dams challenged during pregnancy showed normal fetal development and placental architecture, effectively preventing the fetal loss and pathology observed in control groups. Further immunological analysis revealed that this protection was associated with a balanced immune response at the maternal-fetal interface, characterized by modulated expression of key transcriptional regulators. Specifically, we observed upregulated FOXP3 expression, indicating enhanced regulatory T cell responses critical for maintaining pregnancy tolerance [34], alongside downregulated RORγt expression, suggesting restrained Th17-mediated inflammatory responses that could compromise fetal viability [35,36] (Figure S2(A,B)). This coordinated immunomodulatory profile likely contributed to the preservation of placental integrity and fetal development. The capacity to protect against congenital disease, while promoting appropriate immune regulation at this critical interface, addresses a severe and unmet medical need and highlights the vaccine’s potential for profound public health impact [5].

The conserved DEAD-box motif identified in *T. gondii* DDX6 (Figure S1) confirms its classification within a family of RNA helicases fundamental to RNA metabolism. DEAD-box proteins typically function in ATP-dependent RNA unwinding, remodeling of ribonucleoprotein complexes, and translational control [37]. In other systems, DDX6 is a core component of processing bodies (P-bodies) and stress granules, where it plays a pivotal role in mRNA decay, storage, and translational repression by facilitating the assembly of these ribonucleoprotein complexes [38]. Our hypothesis that DDX6 deletion would lead to broad gene expression alterations is firmly supported by its evolutionarily conserved function as a post-transcriptional regulator. The transcriptomic analysis revealed that DDX6 depletion resulted in a striking imbalance, with 554 genes significantly downregulated compared to only 19 upregulated. This suggests that DDX6 may play an important role in maintaining coordinated gene expression in *T. gondii*. The significant downregulation of critical virulence factors, such as rhoptry proteins (ROPs) and surface antigens (SAGs), may partially explain the observed defects in host cell invasion and parasite virulence. Beyond virulence factors, KEGG pathway analysis indicated widespread disruptions in metabolic pathways and signal transduction. Such dysregulation may impair the parasite’s ability to efficiently generate energy, synthesize essential metabolites, and respond to environmental stresses within the host cell, thereby compromising its overall fitness and replicative capacity. In addition, GO and KEGG enrichment analyses identified alterations in pathways associated with intracellular transport and vesicle-mediated processes. These transcriptomic findings are consistent with the fragmented STX6-positive trans-Golgi phenotype observed in the Δddx6 strain and suggest that DDX6 deletion may affect vesicular trafficking and protein sorting required for proper secretion of invasion- and virulence-associated proteins. Such defects may contribute to the impaired gliding motility, reduced host cell invasion efficiency, and attenuated intracellular proliferation observed in the mutant parasites. Collectively, these findings suggest that widespread disruption of gene regulatory and cellular homeostasis pathways may contribute to the severe attenuation phenotype observed *in vivo*. Although the present data support a strong association between DDX6 deletion and widespread transcriptional dysregulation, additional mechanistic studies will be required to determine the direct molecular pathways underlying these phenotypes.

Although the Δ*ddx6* vaccine conferred robust protection against acute RH challenge, the present study primarily evaluated protective efficacy based on survival outcomes. A more comprehensive assessment incorporating quantitative parasite burden together with longitudinal analyses of parasite dissemination and host immune responses would provide additional mechanistic insight into vaccine-mediated protection. Such investigations require dedicated experimental designs with predefined sampling at multiple stages of infection and therefore will be an important focus of our future studies. Beyond these experimental considerations, live-attenuated vaccines inherently raise potential biosafety concerns, including the theoretical risk of reversion to virulence and safety issues in immunocompromised hosts [39,40]. In addition, murine models cannot fully recapitulate the complexity of human toxoplasmosis, immune responses, or placental physiology [41]. Therefore, further evaluation of long-term safety, genetic stability, and vaccine efficacy in additional animal models will be necessary before potential translational application of the Δ*ddx6* vaccine strategy.

In summary, our integrated phenotypic and transcriptomic analysis establishes DDX6 as a high-level regulatory node in *T. gondii*, whose deletion leads to extensive gene expression dysregulation spanning virulence, metabolic, and signaling pathways. This global reprogramming underpins the Δ*ddx6* strain’s unique profile of complete attenuation, robust immunogenicity, and ability to confer sterilizing immunity across diverse disease models, thereby presenting a highly promising strategy for future toxoplasmosis vaccine development.

## Conclusions

In summary, this study successfully developed a novel live-attenuated vaccine candidate, the RH Δ*ddx6* strain, by disrupting a single gene encoding the RNA helicase DDX6 in *T. gondii*. The vaccine demonstrates an ideal combination of complete attenuation, robust immunogenicity, and broad-spectrum efficacy, conferring nearly sterilizing immunity against acute, chronic, and congenital toxoplasmosis in murine models. Transcriptomic profiling revealed that DDX6 acts as a global regulatory node, whose deletion leads to widespread gene dysregulation, providing a molecular basis for the observed safety and efficacy. This work not only presents a promising vaccine candidate but also validates the strategic advantage of targeting master regulators in apicomplexan parasite vaccine development, offering new insights for combating global toxoplasmosis.

## Supplementary Material

Supplementary materials.docx

ARRIVE FILE.pdf

## Data Availability

The datasets supporting the conclusions of this article are included within the article and its supplemental files. The source data (raw numerical data and statistical calculations) in main manuscript and supplementary files is openly available in Figshare (doi.org/10.6084/m9.figshare.31095208).

## Abbreviations

HFF: Human Foreskin Fibroblasts
DMEM: Dulbecco’s Modified Eagle Medium
FBS: Fetal bovine serum
PBS: Phosphate buffered saline
PCR: Polymerase Chain Reaction
qPCR: Quantitative Real-Time PCR
RT-PCR: Reverse Transcription PCR
OD: Optical Density
*T. gondii*: *Toxoplasma gondii*
BSA: Bovine Serum Albumin
PBST: Phosphate-Buffered Saline with Tween-20
EC buffer: Extracellular Buffer
CFDA-SE: Carboxyfluorescein Diacetate Succinimidyl Ester
GD: Gestational Day
DHFR-TS: Dihydrofolate Reductase-Thymidylate Synthase
CDS: Coding Sequence
ICR: Institute of Cancer Research
ELISA: Enzyme-Linked Immunosorbent Assay
SUB: Subtilisin-Like Protease
FOXP3: Forkhead Box P3
RORγt: Retinoic Acid-Related Orphan Receptor γT
ROP: Rhoptry Protein
SAG: Surface Antigen
GRA: Granule Protein
DEAD-box RNA Helicase: Aspartic acid-Glutamic acid-Alanine-Aspartic acid
PV: Parasitophorous Vacuole
STAg: Secreted Toxoplasma Antigen
DEG: Differentially expressed gene

## References

[1] Kim K, Weiss LM. Toxoplasma gondii: the model apicomplexan. Int J Parasitol. 2004;34(3):423–23. doi: 10.1016/j.ijpara.2003.12.009 15003501 PMC3086386

[2] Molan A, Nosaka K, Hunter M, et al. Global status of Toxoplasma gondii infection: systematic review and prevalence snapshots. Trop Biomed. 2019;36(4):898–925. 33597463

[3] Jones JL, Dubey JP. Foodborne toxoplasmosis. Clin Infect Dis. 2012;55(6):845–851. doi: 10.1093/cid/cis508 22618566

[4] Dian S, Ganiem AR, Ekawardhani S. Cerebral toxoplasmosis in HIV-infected patients: a review. Pathog Glob Health. 2023;117(1):14–23. doi: 10.1080/20477724.2022.2083977 35694771 PMC9848325

[5] Bollani L, Auriti C, Achille C. Congenital toxoplasmosis: the state of the art. Front Pediatr. 2022;10:894573. doi: 10.3389/fped.2022.894573 35874584 PMC9301253

[6] Deganich M, Boudreaux C, Benmerzouga I. Toxoplasmosis infection during pregnancy. Trop Med Infect Dis. 2022;8(1):3. doi: 10.3390/tropicalmed8010003 36668910 PMC9862191

[7] Buxton D, Innes EA. A commercial vaccine for ovine toxoplasmosis. Parasitology. 1995;110 Suppl(S1):S11–16. doi: 10.1017/S003118200000144X 7784124

[8] Dziadek B, Gatkowska J, Grzybowski M. Toxoplasma gondii: the vaccine potential of three trivalent antigen-cocktails composed of recombinant ROP2, ROP4, GRA4 and SAG1 proteins against chronic toxoplasmosis in BALB/c mice. Exp Parasitol. 2012;131(1):133–138. doi: 10.1016/j.exppara.2012.02.026 22445587

[9] Zhang X, Yuan H, Mahmmod YS. Insight into the current Toxoplasma gondii DNA vaccine: a review article. Expert Rev Vaccines. 2023;22(1):66–89. doi: 10.1080/14760584.2023.2157818 36508550

[10] Brito C, Lourenco C, Magalhaes J, et al. Nanoparticles as a delivery System of antigens for the development of an effective vaccine against Toxoplasma gondii. Vaccines (Basel). 2023;11(4):733. doi: 10.3390/vaccines11040733 37112645 PMC10142924

[11] Hunter CA, Sibley LD. Modulation of innate immunity by Toxoplasma gondii virulence effectors. Nat Rev Microbiol. 2012;10(11):766–778. doi: 10.1038/nrmicro2858 23070557 PMC3689224

[12] Wang JL, Zhang N-Z, Li T-T. Advances in the development of anti-Toxoplasma gondii vaccines: challenges, opportunities, and perspectives. Trends Parasitol. 2019;35(3):239–253. doi: 10.1016/j.pt.2019.01.005 30718083

[13] Buxton D, Thomson K, Maley S, et al. Vaccination of sheep with a live incomplete strain (S48) of Toxoplasma gondii and their immunity to challenge when pregnant. Vet Rec. 1991;129(5):89–93. doi: 10.1136/vr.129.5.89 1926725

[14] Burrells A, Benavides J, Cantón G. Vaccination of pigs with the S48 strain of Toxoplasma gondii–safer meat for human consumption. Vet Res. 2015;46(1):47. doi: 10.1186/s13567-015-0177-0 25928856 PMC4415212

[15] Shen B, Brown K, Long S, et al. Development of CRISPR/Cas9 for efficient genome editing in Toxoplasma gondii. Methods Mol Biol. 2017;1498:79–103. 27709570 10.1007/978-1-4939-6472-7_6

[16] Li J, Kang Y, Wu Z-X. Live-attenuated PruDeltagra72 strain of Toxoplasma gondii induces strong protective immunity against acute and chronic toxoplasmosis in mice. Parasit Vectors. 2024;17(1):377. doi: 10.1186/s13071-024-06461-9 39237959 PMC11378421

[17] Wang C, Fu S, Yu X. Toxoplasma WH3 Deltarop18 acts as a live attenuated vaccine against acute and chronic toxoplasmosis. NPJ Vaccines. 2024;9(1):197. doi: 10.1038/s41541-024-00996-9 39443531 PMC11500380

[18] Guo Q, Guo X, Ji N. Role of 6-phosphogluconate dehydrogenase enzyme 1 in growth and virulence of Toxoplasma gondii and development of attenuated live vaccine. Microb Biotechnol. 2023;16(10):1957–1970. doi: 10.1111/1751-7915.14324 37556171 PMC10527188

[19] Zhang H, Ji N, Su S. Metabolic crosstalk between the mitochondrion and the nucleus is essential for Toxoplasma gondii infection. Commun Biol. 2025;8(1):384. doi: 10.1038/s42003-025-07823-4 40050648 PMC11885449

[20] Fuller-Pace FV. DExD/H box RNA helicases: multifunctional proteins with important roles in transcriptional regulation. Nucleic Acids Res. 2006;34(15):4206–4215. doi: 10.1093/nar/gkl460 16935882 PMC1616952

[21] Samir P, Kanneganti TD. DEAD/H-Box helicases in immunity, inflammation, cell differentiation, and cell death and disease. Cells. 2022;11(10):1608. doi: 10.3390/cells11101608 35626643 PMC9139286

[22] Hausmann S, Gonzalez D, Geiser J, et al. The DEAD-box RNA helicase RhlE2 is a global regulator of Pseudomonas aeruginosa lifestyle and pathogenesis. Nucleic Acids Res. 2021;49(12):6925–6940. doi: 10.1093/nar/gkab503 34151378 PMC8266600

[23] El Mortaji L, Aubert S, Galtier E. The sole DEAD-Box RNA helicase of the gastric pathogen Helicobacter pylori is essential for colonization. Mbio. 2018;9(2). doi: 10.1128/mBio.02071-17 PMC5874925 29588407

[24] Cherry AA, Ananvoranich S. Characterization of a homolog of DEAD-box RNA helicases in Toxoplasma gondii as a marker of cytoplasmic mRNP stress granules. Gene. 2014;543(1):34–44. doi: 10.1016/j.gene.2014.04.011 24709106

[25] Ripin N, Macedo de Vasconcelos L, Ugay DA, et al. DDX6 modulates P-body and stress granule assembly, composition, and docking. J Cell Biol. 2024;223(6). doi: 10.1083/jcb.202306022 PMC10978804 38536035

[26] Nguyen TD, de Kesel M, Bigaignon G. Detection of Toxoplasma gondii tachyzoites and bradyzoites in blood, urine, and brains of infected mice. Clin Diagn Lab Immunol. 1996;3(6):635–639. doi: 10.1128/cdli.3.6.635-639.1996 8914751 PMC170423

[27] Ren B, Haase R, Patray S. Architecture of the Toxoplasma gondii apical polar ring and its role in gliding motility and invasion. Proc Natl Acad Sci USA. 2024;121(46):e2416602121. doi: 10.1073/pnas.2416602121 39514309 PMC11573658

[28] Wang J, Wang Y, Zhang H. Evaluation of protective efficacy of recombinant Toxoplasma gondii DDX39 protein vaccine against acute and chronic T. gondii infection in mice. Acta Trop. 2024;260:107442. doi: 10.1016/j.actatropica.2024.107442 39461580

[29] Weber R, Chang CT. Human DDX6 regulates translation and decay of inefficiently translated mRNAs. Elife. 2024;13. doi: 10.7554/eLife.92426 PMC11239181 38989862

[30] Shen Y, Zheng B, Sun H. A live attenuated RHDeltaompdcDeltauprt mutant of Toxoplasma gondii induces strong protective immunity against toxoplasmosis in mice and cats. Infect Dis Poverty. 2023;12(1):60. doi: 10.1186/s40249-023-01109-9 37322556 PMC10266960

[31] Xia N, Zhou T, Liang X. A lactate fermentation mutant of Toxoplasma stimulates protective immunity against acute and chronic toxoplasmosis. Front Immunol. 2018;9:1814. doi: 10.3389/fimmu.2018.01814 30147689 PMC6096001

[32] Zhang HS, Cao H, Li C-X. Immunization with live-attenuated RHDeltahad2a strain confers partial protective immunity against acute and chronic infection of Toxoplasma gondii in mice. Pathogens. 2024;13(2):121. doi: 10.3390/pathogens13020121 38392859 PMC10892008

[33] Chu KB, Quan FS. Advances in Toxoplasma gondii vaccines: current strategies and challenges for vaccine development. Vaccines (Basel). 2021;9(5):413. doi: 10.3390/vaccines9050413 33919060 PMC8143161

[34] Jongert E, Roberts CW, Gargano N, et al. Vaccines against Toxoplasma gondii: challenges and opportunities. Mem Inst Oswaldo Cruz. 2009;104(2):252–266. doi: 10.1590/S0074-02762009000200019 19430651

[35] Xu L, Yu Y, Sang R. Inonotus obliquus polysaccharide protects against adverse pregnancy caused by Toxoplasma gondii infection through regulating Th17/Treg balance via TLR4/NF-kappaB pathway. Int J Biol Macromol. 2020;146:832–840. doi: 10.1016/j.ijbiomac.2019.10.051 31726135

[36] Zhang H, Hu X, Liu X. The Treg/Th17 imbalance in Toxoplasma gondii-infected pregnant mice. Am J Reprod Immunol. 2012;67(2):112–121. doi: 10.1111/j.1600-0897.2011.01065.x 21923716

[37] Weil D, Piton A, Lessel D, et al. Mutations in genes encoding regulators of mRNA decapping and translation initiation: links to intellectual disability. Biochem Soc Trans. 2020;48(3):1199–1211. doi: 10.1042/BST20200109 32412080 PMC7329352

[38] Lumb JH, Li Q, Popov LM. DDX6 represses aberrant activation of interferon-stimulated genes. Cell Rep. 2017;20(4):819–831. doi: 10.1016/j.celrep.2017.06.085 28746868 PMC5551412

[39] Zhang Y, Li D, Lu S, et al. Toxoplasmosis vaccines: what we have and where to go? NPJ Vaccines. 2022;7(1):131. doi: 10.1038/s41541-022-00563-0 36310233 PMC9618413

[40] Liu Q, Singla LD, Zhou H. Vaccines against Toxoplasma gondii: status, challenges and future directions. Hum Vaccin Immunother. 2012;8(9):1305–1308. doi: 10.4161/hv.21006 22906945 PMC3579912

[41] Seo HH, Han H-W, Lee S-E. Modelling Toxoplasma gondii infection in human cerebral organoids. Emerg Microbes Infect. 2020;9(1):1943–1954. doi: 10.1080/22221751.2020.1812435 32820712 PMC7534270

