# Supplementary material for: Live-attenuated Toxoplasma gondii RHΔddx6 mutant establishes comprehensive immunity against acute, chronic, and congenital toxoplasmosis
Source: Virulence. 2026 Aug 7;17(1):2713823. doi: 10.1080/21505594.2026.2713823 (PMC13456505; doi:10.1080/21505594.2026.2713823)
Supplement: Supplementary file 1 [file KVIR_A_2713823_SM7096.pdf]

NOTE: Please save this file locally before filling in the table, DO NOT work on the file within your internet browser as changes will not be saved. Adobe Acrobat Reader (available free here) is recommended for completion.

The ARRIVE guidelines 2.0: author checklist

The ARRIVE Essential 10

These items are the basic minimum to include in a manuscript. Without this information, readers and reviewers cannot assess the reliability of the findings.

Item		Recommendation	Section/line number, or reason for not reporting
**Study design**	1	For each experiment, provide brief details of study design including:	
		a. The groups being compared, including control groups. If no control group has been used, the rationale should be stated.	Control groups are indicated in each figure
		b. The experimental unit (e.g. a single animal, litter, or cage of animals).	Figure legends indicate sample size
**Sample size**	2	a. Specify the exact number of experimental units allocated to each group, and the total number in each experiment. Also indicate the total number of animals used.	Sample sizes per group were indicated. A total of 350 mice were evaluated
		b. Explain how the sample size was decided. Provide details of any *a priori* sample size calculation, if done.	Determined based on previous invesgators' experience
**Inclusion and exclusion criteria**	3	a. Describe any criteria used for including and excluding animals (or experimental units) during the experiment, and data points during the analysis. Specify if these criteria were established *a priori*. If no criteria were set, state this explicitly.	Materials and Method section describe murine inclusion criteria for in vivo experiments. Lines 127-129
		b. For each experimental group, report any animals, experimental units or data points not included in the analysis and explain why. If there were no exclusions, state so.	No exclusion criteria for animals
		c. For each analysis, report the exact value of *n* in each experimental group.	Is reported in each figure legend
**Randomisation**	4	a. State whether randomisation was used to allocate experimental units to control and treatment groups. If done, provide the method used to generate the randomisation sequence.	Materials and Method section. Lines 129-131
		b. Describe the strategy used to minimise potential confounders such as the order of treatments and measurements, or animal/cage location. If confounders were not controlled, state this explicitly.	As above
**Blinding**	5	Describe who was aware of the group allocation at the different stages of the experiment (during the allocation, the conduct of the experiment, the outcome assessment, and the data analysis).	The researcher(s) performing randomization, and preparation of injectant were aware of group assignment.
**Outcome measures**	6	a. Clearly define all outcome measures assessed (e.g. cell death, molecular markers, or behavioural changes).	Materials and Method section
		b. For hypothesis-testing studies, specify the primary outcome measure, i.e. the outcome measure that was used to determine the sample size.	Described in each figure legend
**Statistical methods**	7	a. Provide details of the statistical methods used for each analysis, including software used.	Materials and Method section.Lines 411-418
		b. Describe any methods used to assess whether the data met the assumptions of the statistical approach, and what was done if the assumptions were not met.	As above
**Experimental animals**	8	a. Provide species-appropriate details of the animals used, including species, strain and substrain, sex, age or developmental stage, and, if relevant, weight.	Materials and Method section.Lines 127-129
		b. Provide further relevant information on the provenance of animals, health/immune status, genetic modification status, genotype, and any previous procedures.	NA
**Experimental procedures**	9	For each experimental group, including controls, describe the procedures in enough detail to allow others to replicate them, including:	Described in the Materials and Method section
		a. What was done, how it was done and what was used.	
		b. When and how often.	
		c. Where (including detail of any acclimatisation periods).	
		d. Why (provide rationale for procedures).	
**Results**	10	For each experiment conducted, including independent replications, report:	Described in the Materials and Method section
		a. Summary/descriptive statistics for each experimental group, with a measure of variability where applicable (e.g. mean and SD, or median and range).	
		b. If applicable, the effect size with a confidence interval.	
