# Supplementary material for: Live-attenuated Toxoplasma gondii RHΔddx6 mutant establishes comprehensive immunity against acute, chronic, and congenital toxoplasmosis
Source: Virulence. 2026 Aug 7;17(1):2713823. doi: 10.1080/21505594.2026.2713823 (PMC13456505; doi:10.1080/21505594.2026.2713823)
Supplement: Supplementary file 2 [file KVIR_A_2713823_SM7097.docx]

**Figure S1**

**Multiple sequence alignment of DDX6 protein across diverse eukaryotic species.**

Protein sequences of DDX6 homologs from *Saccharomyces cerevisiae* (strain YJM14), *Homo sapiens*, *Mus musculus*, *Plasmodium falciparum* (strain 3D7), and *T. gondii* (RH strain) were aligned. The conserved DEAD motif, characteristic of DEAD-box RNA helicases, is highlighted in red boxes across all species, demonstrating the evolutionary

conservation of this functional domain.

**Figure S2**

**FOXP3 and RORγt mRNA expression levels in placental tissues at GD13.5.**

**A** Relative mRNA expression levels of FOXP3, a key transcription factor for regulatory T cells. **B** Relative mRNA expression levels of RORγt, a critical regulator of Th17 cell differentiation. Placental tissues were collected at gestational day 13.5 (GD13.5) from the following groups: NC (normal control), RH (challenged with *T. gondii*RH strain), Vac (immunized with Δ*ddx6* strain), and Vac+RH (immunized and challenged). Gene expression was quantified by RT-qPCR and normalized to reference genes. Data are presented as mean ± SD from three independent experiments. Statistical significance was determined by one-way ANOVA with Tukey's post-hoc test (**P* < 0.05, ***P* < 0.01).

**Figure S3**

**Functional enrichment analysis of DEGs between** Δ***ddx6* and RH strains.**

**A** KEGG pathway enrichment analysis of DEGs. The histogram shows the number of genes enriched in major KEGG pathways, including human diseases, metabolism, genetic information processing, and environmental information processing. Pathways are categorized and color-coded by functional classification. **B** Gene Ontology (GO) term analysis of DEGs. The bars represent the number of up-regulated (blue) and down-regulated (orange) genes in three GO categories: biological process, molecular function, and cellular component. The analysis reveals significant alterations in metabolic pathways and cellular component organization in Δ*ddx6* mutants compared to the wild-type RH strain, indicating broad functional impacts of DDX6 deletion on parasite biology.
